# School absenteeism in autistic children and adolescents: A scoping review

**DOI:** 10.1177/13623613231217409

**Published:** 2023-12-30

**Authors:** Viviann Nordin, Maud Palmgren, Anna Lindbladh, Sven Bölte, Ulf Jonsson

**Affiliations:** 1 Center of Neurodevelopmental Disorders (KIND), Centre for Psychiatry Research; Department of Women’s and Children’s Health, Karolinska Institutet & Stockholm Health Care Services, Region Stockholm, Stockholm, Sweden; 2 Child and Adolescent Psychiatry, Stockholm Health Care Services, Region Stockholm, Stockholm, Sweden; 3 Curtin Autism Research Group, Curtin School of Allied Health, Curtin University, Perth, Australia; 4 Department of Medical Sciences, Child and Adolescent Psychiatry, Uppsala University, Uppsala, Sweden

**Keywords:** absenteeism, autism, dropout, interventions, prevalence, risk factor, school refusal

## Abstract

School absenteeism is a major societal problem, with a range of potential adverse long-term consequences. This scoping review aimed to provide a comprehensive overview of the research on school absenteeism in autistic children and adolescents, expose important gaps in the literature, and explore possibilities for future systematic reviews. Five relevant databases were searched systematically from inception to June 2023, yielding a total of 46 eligible reports from 42 separate studies. All studies were conducted in high-income countries, and most were published in the last decade. Three major themes emerged: occurrence, contextual factors, and interventions. The results of large-scale population-based studies clearly suggested that autistic children and adolescents were absent from school more often than their non-autistic peers, which partly was attributable to co-occurring conditions. Bullying also emerged as a potential risk factor. Only a few preliminary studies were available on targeted interventions, emphasizing the need for more robust studies. More research is also needed on the mechanisms leading to and maintaining school absenteeism in this group of learners. Overall, the diversity of research questions, methods, and definitions used in this body of research suggests that systematic reviews with narrow focus on a few key questions may still be premature.

**Lay abstract:**

Autistic children and teenagers are, on average, absent from school more than their peers. The aim of this review was to provide an overview of the research on absence from school in autistic learners in primary and secondary school, to help guide future research. We sifted through 4632 reports and found 42 studies with a focus on school absence and autism. We looked at how, when, and where the studies were conducted. We also summarized the results and outlined how absence was measured in the studies. Absence from school may lead to problems later in life, like incomplete education and unemployment. It is therefore important to know how common this problem is among autistic learners, what the reasons may be, and what type of support they need. The studies were from high-income countries and were mainly published in the last 10 years. Studies based on school registers from the United States and the United Kingdom clearly showed that children and teenagers with autism had higher risk of school absence than those without autism. Absence was often linked to problems with mental health or additional neurodevelopmental conditions. Several studies also showed that absence in autistic children and adolescents was related to problems in school, like bullying or lack of knowledge about autism. Support programs were only evaluated in a few studies with a small number of study participants. We conclude that more research is needed to better understand why autistic learners are absent and what they need to thrive in school.

## Introduction

Education is a basic human right, preparing children and adolescents (hereafter called children) for their future life and active citizenship. Still, it has been estimated that one out of five children are out of school (UNESCO, 2020). While children growing up in some parts of the world may be deprived of education due to poverty, political conflict, war, and gender inequalities, school absenteeism and premature school dropout are major societal challenges even in countries where public education is available for all. In addition, school closures during the COVID-19 pandemic recently disrupted education for children throughout the world (UNESCO, 2022), possibly aggravating the situation.

Studies suggest that absenteeism interferes with everyday functioning in school, with peers, and within the family (Kearney, 2022). Frequent absence from school increases the risk of low academic achievement, risk behaviors, substance use, and mental health problems (Epstein et al., 2020; Gottfried, 2009; Kearney, 2008). In the longer term, a range of adverse adult outcomes may follow, such as ill-health, economic deprivation, marital problems, and unemployment (Kearney, 2008). Complex and multifaceted underlying causal mechanisms have been outlined in previous research, including mental and physical health problems, adverse childhood experiences, low parent-school involvement, and socioeconomic factors (Crouch et al., 2019; Finning et al., 2019; Gubbels et al., 2019; Hancock et al., 2021; John et al., 2022; Kearney, 2008; Sosu et al., 2021). School-related factors are also likely to play an important role, including teachers’ classroom management and bullying victimization (Havik et al., 2015; Olweus, 2013; Vidourek et al., 2016).

To add to the complexity, a range of different types of absence have been described in the literature (Heyne et al., 2019, 2020; Kearney et al., 2019a, 2019b). Distinctions have been made between authorized (e.g. due to illness and health appointment) and non-authorized (e.g. school refusal and truancy) absence (Kearney et al., 2019a). Other frequently used terms include school refusal (due to emotional difficulties), school withdrawal (parent-initiated), exclusion (school initiated), truancy (unauthorized), and school dropout (premature departure from school) (Kearney et al., 2019a). Several terms are also used to delimit more severe forms of absence, including chronic absenteeism (missing 15 days in 1 year) (U.S. Department of Education, 2019) and persistent absence (missing over 10% of sessions) (United Kingdom Department for Education, 2021). The lack of consensus related to definitions and categorizations have recently been raised as one of the key challenges in this research field (Heyne et al., 2019, 2020).

In addition, the evidence suggests that the effects of available intervention programs are modest. A recent meta-analysis included data from 22 controlled intervention studies (Eklund et al., 2022). The three main forms of intervention—behavioral interventions, family-school partnerships, and academic interventions—had small positive effects on school attendance. The authors discuss the heterogeneity and quality issues concerning many of the studies. A systematic review and meta-analysis of interventions for school absenteeism in children with anxiety—mainly cognitive-behavioral approaches—found a significant effect on school attendance, but not on anxiety (Maynard et al., 2018).

Students with disabilities and special education needs are particularly vulnerable (Gottfried et al., 2019; Stromberg et al., 2022). While this group of students can be expected to have elevated levels of both unauthorized and authorized absence related to their disability and co-occurring health conditions, the pattern might be quite specific for different disabilities. Autistic children constitute a distinct group of learners that often find the school environment particularly challenging due to insufficient accommodations (L. Anderson, 2020; Dargue et al., 2022; Leifler et al., 2021). Some of the factors leading to and maintaining school absenteeism might be specific to autistic children (Adams et al., 2022), which in turn may have implications for how targeted interventions should be designed. For instance, bullying victimization is more common in this population than among non-autistic peers (Maiano et al., 2016). The high prevalence of co-existing neurodevelopmental conditions, mental disorders, and somatic health conditions (Hossain et al., 2020; Pan et al., 2021; Thapar et al., 2017) are also likely to impact on school absenteeism.

Recent population-based studies on neurodevelopmental conditions suggest elevated levels of school absenteeism in autistic children compared with their peers in the United States and the United Kingdom (K. P. Anderson, 2021; John et al., 2022). Still, no comprehensive overview of the accumulated research on school absenteeism and autism is currently available to guide upcoming research initiatives. We deemed that a scoping review would be an appropriate first step, with an aim to provide a broad overview of all research published to date on all forms of absence (e.g. chronic absenteeism/persistent absence, authorized absence, exclusion, dropout, and withdrawal) from primary and secondary school in children on the autism spectrum. The objectives of this scoping review were to

1. Provide an overview of the accumulated research in this field,
2. Expose important gaps in the literature, and
3. Explore possibilities for future systematic reviews with narrower review questions.

## Method

### Design

The present scoping review is part of a project aiming to provide an overview of the overall literature on school absenteeism in children with neurodevelopmental conditions. The review has been reported in accordance with the PRISMA Extension for Scoping Reviews (Tricco et al., 2018). The review was not pre-registered, and the protocol has not been published. There was no community involvement.

### Eligibility criteria

#### Population

The study population included children in primary and secondary school age with a diagnosis on the autism spectrum as defined by the current or previous editions of the *Diagnostic and Statistical Manual of Mental Disorders* (*DSM*) or the International Classification of Diseases (ICD). Conditions were identified by clinical assessment, validated instruments based on the above classifications, parental report, or retrieval from administrative registers. Studies focusing on diverse conditions and disabilities were excluded, unless data were reported separately for the autistic participants.

#### Concept

Any form of absence from school in childhood or adolescence, including, but not limited to, chronic absenteeism, school refusal, exclusion, dropout, and withdrawal.

#### Context

Any type of primary or secondary school.

#### Design

All research designs.

#### Publication type

Original research papers published in English in peer-reviewed journals. Dissertations, books, review papers, theoretical papers, and governmental reports were not included.

### Search strategy

A literature search was performed in the following databases: Cochrane (Wiley), Medline (Ovid), PsychInfo (Ovid), Web of Science Core Collection, and ERIC (ProQuest). The search strategy was developed in Medline (Ovid) in collaboration with librarians at the Karolinska Institutet University Library. For each search concept, Medical Subject Headings (MeSH terms) and free text terms were identified. Two distinct blocks were combined: the population (terms related to neurodevelopmental conditions) and the concept (terms related to school absenteeism). The search was then translated into the other databases. The strategies were peer reviewed by another librarian prior to execution. No language restriction was applied. Databases were searched from inception, and the search was last updated on 9 June 2023. The update and de-duplication were done using established methods (Bramer & Bain, 2017; Bramer et al., 2016). The full search strategies for all databases are available in Supplemental Appendix A.

### Study selection

All references were screened independently by two review authors using EndNote (for the initial stage) and Rayyan (for an updated search). Publications found to be of potential relevance by at least one of the review authors were obtained in full text and assessed for eligibility independently by both. Ultimately, 12.5% of the citations screened were obtained in full text. Disagreements at the full-text stage were solved by consensus during regular meetings. If necessary, a third review author was consulted. The two assessors disagreed on seven full-text reports, of which four were solved by consensus and three after discussion with a third assessor.

### Data charting process

Guided by our objectives, a data extraction sheet was created in excel. In a pilot test, three members of the team extracted information from five reports and thereafter collaboratively revised the sheet for usability. Data were thereafter extracted from eligible studies by one review author. The data were double checked by a second review author. A third review author checked the integrity of key information during the synthesis. One item (funding) was added during the data extraction phase.

### Data items

The extracted information pertained to study characteristics (year of publication, country, diagnosis, age span, gender distribution, type of sample, sample size, design, research questions/objectives, definitions and concepts related to absence, data sources, and funding) and quantitative and qualitative data related to absenteeism (e.g. prevalence or other estimates of occurrence, contextual factors associated with absenteeism, intervention outcome, type of interventions and their main components, and experiences related to absenteeism).

### Synthesis

To identify major themes in the literature, the research questions/objectives of each individual study were extracted. We also familiarized ourselves with the studies by reading each report carefully, to identify additional questions of relevance that were addressed but not explicitly stated in the reports. One review author coded the topic(s) of each report, based on the research questions/objectives identified. More than one topic could be coded for each report/study. The coded topics may or may not be identical to the research questions as stated by the authors of the original studies. An inductive approach was then used to identify major themes among the topics addressed in the reports. The coded topics and themes were reviewed, refined, and named by three of the authors in an iterative process. To further enhance readability, the information subsumed under each theme was organized under subheadings based on study design and/or specific topics. For each individual study, information related to key results, samples, and design were briefly summarized in the text. Characteristics of the individual studies were presented in tables and summarized across studies.

The key findings related to each of the objectives of our scoping review were synthesis across the whole body of research and within the major themes. To summarize the overview of the research, general patterns of results were derived based on the number of studies published, the specific topics addressed, the direction of results, and the sample sizes of relevant studies. Gaps in the literature were inferred by considering neglected areas of research, identifying less represented demographics, and pinpointing topics addressed in few or preliminary studies. Preconditions for future systematic reviews were determined based on the number of eligible studies for major potential review questions, the sample sizes of these studies, and their heterogeneity in terms of definitions of concepts, exposures, designs, measures, statistical methods, populations, comparison groups, and interventions. The synthesis was drafted by one of the authors, and subsequently discussed and revised together with two additional review authors.

## Results

### Study selection

**Figure 1.**
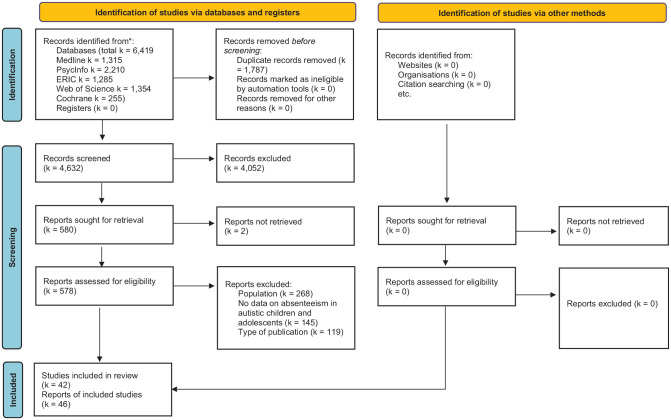
PRISMA 2020 flow diagram.

After removal of duplicates, 4632 records were screened. From these, 578 full-text documents were assessed for eligibility. A total of 46 reports based on 42 unique studies met the eligibility criteria. The remaining 532 full-text documents were excluded (see Supplemental Appendix B). Results from the following studies were presented in more than one report: an Australian study by Bitsika and colleagues (Bitsika et al., 2021; Bitsika, Heyne, & Sharpley, 2022; Bitsika, Sharpley, & Heyne, 2022); an Australian study by Adams and co-workers (Adams, 2022; Adams et al., 2022); and a Norwegian study by Munkhaugen and colleagues (Munkhaugen et al., 2017, 2019). The study selection is outlined in Figure 1.

### Study characteristics

The included studies were conducted in the United States (*k* = 14), the United Kingdom (*k* = 13), Japan (*k* = 7), Australia (*k* = 3), Sweden (*k* = 3), the Netherlands (*k* = 1), and Norway (*k* = 1). While the oldest publication was from 1991 (Kurita, 1991), most reports (*k* = 38) were published during the last 10 years. Eighteen of the studies were supported by external funding from foundations or research councils, while the remaining either did not receive external funding (*k* = 12) or did not include a statement about funding (*k* = 12). Participants were predominantly male, although the sex ratio was not always reported. The age span ranged from 2 to 21 years, with most of the studies focusing on ages 8–17. Information on school absenteeism was mainly based on parent reports or school attendance data. Only a few studies included information provided by the children themselves (Tables 1 to 3).

**Table 1.** Characteristics of studies reporting on occurrence of school absenteeism.

Author (year) Country	Autistic students, *n*	Participants, total *N*	Sample selection	Age, years	Data source	Topic
Adams (2022)^a^ Australia	106	106	Invited caregivers	Mean age 11.8 (SD 3.1)	Online parent survey including the SNACK	Absence
K. P. Anderson (2021) USA	Student-year observations: 92,949	Student-year observations: total 6,810,430	Special education students	Not reported	5 years of demographic data, attendance records, and disciplinary reports	Absence Exclusion
Chen et al. (2016) USA	163	Approximately 58,000	One public school district	Grade 7–9	Longitudinal (for one school year) population-based study; administrative school attendance data	Truancy
Crump et al. (2013) USA	94	22,730	One school district	Grade 2–11	School performance and absenteeism from school records + health status from parental survey	Absence
Fleming et al. (2020)^a^ UK	10,814	766,244	Population-based study; children in primary, secondary, or special schools	4–19	Linking of data from education and health databases	Absence Exclusion
Hatton et al. (2018) UK	93,710	6,166,565	Population-based study; children with special educational needs	5–15	Statistics on school absences and exclusions from the Department for Education	Absence Exclusions
John et al. (2022)^a^ UK	7055	414,637	Population-based study; children in state-funded schools	7–16	Linking of routinely collected healthcare data on neurodevelopmental diagnoses and data on school attendance and exclusion	Absence Exclusion
Kurita (1991)^a^ Japan	110 on the autism spectrum ± ID	135 (ID and not autism 25)	Guidance center, consecutive cases	12–17	Clinical evaluation + parent questionnaires	Absence
Lee et al. (2008) USA	483	65,755	National Survey of Children’s Health	3–17	Parent-reported data	Absence
Melvin et al. (2023) Australia	321	629	Invited schools and parents; 90.5% special schools, 9.5% mainstream; students with parent-reported ID	5–18	Parent-reported diagnosis; absence data from school administration	Absence
Munkhaugen et al. (2017) Norway	78	216	Mainstream classes; comparison group of typically developing peers	9–16	Absence reported by teachers	Absence
Stromberg et al. (2022) USA	1502	50,138	Nationally representative survey	6–17	Questionnaires to caregivers for randomly selected children	Absence
Takara and Kondo (2014) Japan	70	430	Adult outpatients at a psychiatric clinic	18–87	Retrospective recall	Absence
Tani et al. (2012) Japan	99	668	Adult outpatients with suspected social communicative problems	>18	Retrospective analysis of clinical reports	Absence
Totsika et al. (2020)^a^ UK	486	486	Invited caregivers	2–18	Parental report non-attendance, and online parent questionnaires including the SNACK	Absence

SD: standard deviation; SNACK: School Non-Attendance ChecKlist; ID: intellectual disability.

a Included also in Table 2.

**Table 2.** Characteristics of studies reporting on contextual factors related to school absenteeism.

Author (publication year) Country	Autistic students, *n*	Participants, total *N*	Sample selection	Age, years	Data source	Topic
Adams (2022)^a^ Australia	106	106	Invited caregivers	Mean age 11.8 (SD 3.1)	Online parent questionnaires, including the SNACK	Risk factors
Adams et al. (2022) Australia	121	121	Invited caregivers	Mean age 11.9 (SD 6.0)	Online parent questionnaires including the SRAS-R-P	Assessment tool
L. Anderson (2020) Sweden	1799	1799	Invited parents	6–21	Web-based parent survey	Parent perspectives
Ashburner et al. (2019) USA	89	89	Invited caregivers; diagnosis on the autism spectrum, not ID	11–16	Parent and student questionnaires, and semi-structured interview with parents	Bullying
Bitsika et al. (2021) Australia	67	67	Invited caregivers; boys in mainstream classes	7–18	Parent and child questionnaires	Bullying
Bitsika, Heyne, & Sharpley (2022) Australia	58	58	As in Bitsika et al. (2021)	5–16	As in Bitsika et al. (2021)	Resilience
Bitsika, Sharpley, & Heyne (2022) Australia	71	71	As in Bitsika et al. (2021)	6–18	As in Bitsika et al. (2021)	Anxiety
Brouwer-Borghuis et al. (2019)^b^ The Netherlands	18	30	Adolescents with internalizing and/or neurodevelopmental conditions	12–17	Review of files	Bullying
Feldman et al. (2015) USA	46	108	Student with significant disabilities, enrolled in general high school classes	14–20	Observation	Time spent in class; proximity to peers
Fleming et al. (2020)^a^ UK	10,814	766,244	Population-based study; children in primary, secondary, or special schools	4–19	Linking of data from education and health databases	Multimorbidity
Foster and Pearson (2012) USA	395	395	Nationally representative sample of youth with autism in special education	13–16 (year 2000); 20–23 (year 2007)	Data from school authorities, and parent telephone interview	Settings (inclusive or not)
Jarbou et al. (2022) USA	120	120	Special education schools	Average: 6	Attendance history, and demographic and individual characteristics	Prediction
John et al. (2022)^a^ UK	7055	414,637	Population-based study; children in state-funded schools	7–16	Linking of routinely collected healthcare data and data on school attendance and exclusion	Co-occurring conditions, age, sex differences
Kouroupa et al. (2023) UK	82% of children from 809 families	Children from 809 families	Invited parents to children with autism and/or ID	5–15	Online parent survey	School closure
Kurita (1991)^a^ Japan	110 on the autism spectrum ± ID	135	Guidance center, consecutive cases	12–17	Clinical evaluation and parent questionnaires	School refusal in autism and/or ID
Lissack and Boyle (2022) UK	289 parents; autism suspected in 51% of children	289 or more	Invited parents of children with school non-attendance	5–16	Online parent survey	Parental view on support from school
Matsuura et al. (2020) Japan	9	175	Patients at medical clinics	8–18	Medical records + questionnaires	Quality of life
Mattson et al. (2022) USA	Preschool: 20; Elementary school: 54	74	Private special education preschool and elementary school	3–12	School attendance data, and assessment of adaptive behavior and cognitive ability	Age; adaptive skills
McClemont et al. (2021) USA	Autism: 36; Autism + ADHD: 31	Children: 154; Parents: 97	Parents recruited by flyers	4–16	Online parent questionnaire	Bullying
Munkhaugen et al. (2019) Norway	62	62	Mainstream classes; participants from Munkhaugen et al. (2017)	9–16	Parent questionnaires	Neuropsychological functioning
Ochi et al. (2020) Japan	94	237	Psychiatric outpatients with school refusal behavior	6–18	Retrospective chart review	Bullying
Paulauskaite at al. (2022) UK	126	158 children with neurodevelopmental conditions	Elective home education	5–15	Online parent survey	Home education
Totsika et al. (2020)^a^ UK	486	486	Invited caregivers	2–18	Parental report on school absence, and online questionnaires including the SNACK	Description of school non-attendance
Wainscot et al. (2008) UK	30	57	Mainstream classes	11–13	Structured interview with students, pedometer, and school-attendance data	Interaction and activity
Vincent et al. (2023) UK	23	23	Online parent groups; children in mainstream classes	School-aged	Interviews with 17 parents	Experiences of lockdown
Widnall et al. (2022) UK	2463	113,286	State secondary schools	11–17	National pupil database	Self-harm

SD: standard deviation; SNACK: School Non-Attendance ChecKlist; SRAS-R-P: School Refusal Assessment Scale–Revised–Parent version; ID: intellectual disability.

a Included also in Table 1.

b Included also in Table 3.

**Table 3.** Characteristics of studies focusing on interventions for school absenteeism.

Author (year) country	Autistic students, *n*	Participants, total *N*	Sample selection	Age, years	Design/data source	Topic
Arvans and LeBlanc (2009) USA	1	1	Autistic boy with migraine and school absence	14	Case study/functional assessment	Behavioral intervention
Brouwer-Borghuis et al. (2019)^a^ The Netherlands	18	30	Adolescents with internalizing and/or neurodevelopmental conditions	12–17	Observational/review of files	Alternative educational program
Byron (2002) UK	1	1	Boy with a social communication disorder (described as autism)	15	Case study/case report	Hypnosis
Guastello et al. (2023) USA	1	1	Gender-neutral individual; home-schooling, online classes	17	Case study/fictionalized case, capturing common themes of autism, OCD, ADHD, and gender diversity	Cognitive behavioral therapy
Hirata et al. (2023) Japan	12	30	Children with developmental difficulties in elementary school	6–12	Qualitative/semi-structured interviews with school counselors	Complaints and type of support
Lambros et al. (2016) USA	36	61	Children with developmental disabilities + mental health problems	4–21	Observational/administrative data and parent, child, and staff questionnaires	School-based mental health outpatient program
Leifler et al. (2022) Sweden	11	13	Mainstream senior high schools	17–20	Qualitative/interviews with students, parents, and school staff	Social skills group training
Melin et al. (2022) Sweden	Not reported	Not reported	Child and adolescent psychiatric service	Not reported	Qualitative/semi-structured interviews with clinicians	Psychiatric care
O’Hagan et al. (2022) UK	3	3	Girls with emotionally based school avoidance; mainstream high school	13–15	Case study/interviews with students, parents, and school staff	Re-engagement in school
Preece and Howley (2018) UK	7	7	Male autistic students with anxiety; local center for children with school non-attendance	14–16	Case study/attendance data, student questionnaires, and interviews	Model of support
Yamada et al. (2020) Japan	1	2	Girls with generalized anxiety disorder/school refusal behavior	10	Case study/case reports	Selective serotonin reuptake inhibitor

OCD: obsessive-compulsive disorder; ADHD: attention-deficit hyperactivity disorder.

a Included also in Table 2.

A wide variety of terms were used to describe absenteeism, including school absenteeism, school absence, school non-attendance, and school refusal. Some studies used terms interchangeably. Studies generally used the terms in a broad sense, including both excused and unexcused absence. Three studies (Adams, 2022; Melvin et al., 2023; Totsika et al., 2020) differentiated between types of non-attendance using the School Non-Attendance ChecKlist (SNACK; Heyne et al., 2019). Most studies measured absence in school days missed during a specified period, often transformed into percentage of missed school time. Other studies gave no description of how absence was measured, or only a vague qualitative description. The terms used to denote problematic absence also varied (e.g. chronic, persistent, continuous, or frequent). The most common definition was 10% of school time, which was used in seven studies (K. P. Anderson, 2021; Hatton, 2018; Jarbou et al., 2022; John et al., 2022; Kouroupa et al., 2023; Mattson et al., 2022; Totsika et al., 2020). Other definitions included 30 days/year, 4 weeks/year, 3 days/20 days, more than 1 week, or 11 days. Several studies did not use a threshold, while some used several different categories (Supplemental Appendix C, Table S1 to S3).

Three major themes emerged: occurrence; contextual factors; and interventions. The individual studies are briefly summarized under each theme, while key findings across studies are presented in Table 4.

**Table 4.** Summary of findings related to the scoping review objectives.

	Overview of the research	Gaps in the literature	Preconditions for future systematic reviews
Overall	• The accumulated body of research in this field is quite sizable and diverse. • Most studies were published during the last decade.	• Studies from low-income countries were lacking. • Few studies explored the autistic children’s lived experience.	• A wide range of definitions of school absenteeism were used.
Occurrence	• Large-scale population-based studies suggest that autistic children are at increased risk for absence from school compared to their non-autistic peers. • These results are corroborated by several smaller studies. • Studies indicate that the increased risk partly is attributable to co-occurring conditions (e.g. neurodevelopmental conditions and common mental disorders). • Studies suggest that autistic children may have elevated levels of both authorized and unauthorized absence.	• The available research was limited to a few countries (predominantly the United Kingdom and the United States). • Sex and age differences are not sufficiently studied.	• Several large-scale studies provide estimates, but definitions and statistical methods varied.
Contextual factors	• A wide range of contextual factors within several different domains were investigated. • The variety of factors linked to school absenteeism in autistic children underscores the complexity of this phenomenon. • Co-occurring conditions and bullying were addressed in several studies and stand out as important focal points for future research and development of interventions.	• Most of the factors were investigated in a single or a few studies, often using relatively small samples, pointing to a general need for replication. • Prospective longitudinal studies will be needed to better understand mechanisms and long-term outcomes.	• The few studies focusing on the same factors are heterogeneous in terms of samples, study designs, definitions, and measurements.
Interventions	• The studies were based on a small number of autistic participants (ranging from 1 to 31). • No comparison groups were used. • The available studies may inform future research. • Targeted intervention programs had some aspects in common, including a focus on building relationships and using an individualized and flexible approach.	• Robust studies evaluating the efficacy and safety of targeted interventions are still lacking. • No studies investigated accommodations in the school environment.	• Available studies are few, preliminary, and heterogeneous (e.g. in terms of interventions, study designs, and outcome measures).

### Occurrence

Here, we include 15 studies presenting different types of estimates or related information on the occurrence of absenteeism. Eight studies used population-based samples, of which five utilized administrative data and school records and three relied on parent report. The remaining studies were based on diverse samples, including clinical samples and parents invited via advertisement (Table 1).

#### Summary of individual studies

##### Population-based studies

The association of school absenteeism and neurodevelopmental and mental disorders was analyzed in two large population-based studies from the United Kingdom (Fleming et al., 2020; John et al., 2022). A population-based cohort study from Wales linked health care data with data on school attendance and exclusion (John et al., 2022). In comparison to children with no registered disorder, children with a diagnosis on the autism spectrum were more likely to have chronic absenteeism (adjusted odds ratio (aOR) = 2.0; 1.9–2.1) and to be excluded from school (aOR = 2.6; 2.4–2.9). Sub-analyses suggested higher risk among those with co-occurring conditions (aOR = 2.5; 2.4–2.7 for chronic absenteeism; aOR = 3.5; 3.2–4.0 for exclusion).

A Scottish study similarly investigated the association of neurodevelopmental disorders with school absence or exclusion (Fleming et al., 2020). Having only an autism spectrum diagnosis was associated with elevated level of absenteeism and exclusion compared to children with no condition, with an adjusted incidence rate ratio (aIRR) of 1.10 (1.08–1.13) for absenteeism and 1.50 (1.30–1.73) for exclusion. The associations were stronger for those with co-occurring conditions, with an estimated aIRR of 2.36 (2.01–2.76) for absenteeism among those with autism and co-existing depression, and 6.04 (4.98–7.33) for exclusion among those with both autism and attention-deficit hyperactivity disorder (ADHD).

Another study from the United Kingdom analyzed reports from school authorities (Hatton, 2018). The level of authorized school absence was higher for children with special needs, including autistic children. Unauthorized absence was only slightly more common than in children without special needs.

A study from the United States analyzing school absence data for special education students (K. P. Anderson, 2021) reported that 19% of children on the autism spectrum were missing at least 10% of school days/year, compared to 14% of students in general education. Two additional studies using administrative and school data (Chen et al., 2016; Crump et al., 2013) included few autistic children in the samples, resulting in uncertain estimates.

The three population-based studies relying on parent report pointed in the same general direction. A report based on the US National Survey of Children’s Health found higher odds of chronic absenteeism in children with autism compared to children with typical development. After adjustment for co-occurring conditions, health status, and psychosocial factors, this association did not remain for the subgroup of younger autistic children with more support needs (Stromberg et al., 2022). Another study from the United States, with a primary focus on quality of life (Lee et al., 2008), reported that autistic children were more likely to miss school days due to illness or injury than children with ADHD and those without any of the conditions.

##### Other samples

Four studies included autistic samples and a comparison group. A Norwegian study (Munkhaugen et al., 2017) included students in mainstream classes with a diagnosis on the autism spectrum. During a 20-day period, school refusal behavior was present in 42.6% of the students with autism compared to 7.1% of randomly chosen non-autistic students. In older children, the pattern shifted to actual absenteeism and not only verbal/physical refusal. In a study of children attending a guidance center in Japan (Kurita, 1991), children on the autism spectrum (with or without concomitant intellectual disability) had higher rate of school refusal behavior compared to a group with intellectual disability without autism. In two retrospective case-control studies of adults visiting psychiatric outpatient clinics in Japan, patients with autism were more likely than other patients to have a history of school non-attendance (Takara & Kondo, 2014; Tani et al., 2012).

A study from the United Kingdom (Totsika et al., 2020) was the first to present a systematic classification of different types of non-attendance using the SNACK. Parents of children on the autism spectrum were invited to an online survey. Persistent absence (missing 10% or more of sessions) occurred among 43% of the students. The most frequent reason for non-attendance was school refusal, followed by non-problematic (authorized) absence which was more common in the group with intellectual disability. School exclusion and school withdrawal each accounted for 9% of days missed. Truancy was rare in this sample. An Australian study (Melvin et al., 2023) reported that the most common causes for non-attendance among children with intellectual disability were illness and health-related appointments. About 10% of autistic students with intellectual disability had at least one absence due to school withdrawal, and 6% due to school refusal. Truancy and exclusion were less common. The pattern was similar for non-autistic children with intellectual disability. Another Australian study (Adams, 2022) reported that children on the autism spectrum missed on average six full days of school each 4 weeks, with school refusal and medical/therapy appointments as the most frequent reasons.

### Contextual factors

A total of 23 studies (26 reports) provided information about potential risk factors and other contextual factors (Table 2). The information was subcategorized into school factors, individual factors, family-related factors, and evaluation of tools for assessment of contextual factors.

#### Summary of individual studies

##### School factors

School placement was addressed in four studies. An online survey from the United Kingdom (Totsika et al., 2020) found an association between mainstream school placement and non-attendance. A study from the United States (Foster & Pearson, 2012) compared outcomes (e.g. not dropping out of high school) for autistic children having more or less of inclusive education. There were no significant differences, and the authors pointed to methodological problems, including finding comparable groups. Another study from the United States (Feldman et al., 2015) observed students with severe disabilities (autism or intellectual disability) in general education high school classes and highlighted that students with disabilities might arrive late and leave early and therefore miss time for instruction and time with peers. Finally, a study from the United Kingdom (Wainscot et al., 2008) found that autistic children in mainstream classes had significantly less physical activity, reported more time alone at breaks and less friends, and were more likely than matched controls to be bullied. However, no differences in school attendance were found.

The learning environment was discussed in five studies, all based on reports from invited parents. In a Swedish online survey (L. Anderson, 2020), parents reported on lack of autism competence among staff, insufficient adaptation of the learning environment, and lack of support. Similar problems were raised in recent studies from the United Kingdom tapping parents’ experiences during the pandemic. Many families experienced a continuation of a stressful situation (Vincent et al., 2023). For some families, however, the level of stress decreased as COVID-19 raised the awareness of the strains met by families where a child is absent from school (Lissack & Boyle, 2022). During the period with online education, children were reportedly feeling more connected to their peers. The parents stressed the importance of a partnership between home and school, to address the problem early, and to stop using the term school refusal. One study found that a reason for selecting elective home education was to avoid exclusion from school (Paulauskaite et al., 2022), while one study (Kouroupa et al., 2023) reported that school attendance problems increased during school closure in a group of children with autism and/or intellectual disability.

Several studies focused on bullying in relation to school absenteeism. An Australian study (Bitsika et al., 2021) found that being bullied was associated with emerging school refusal among autistic children. A study from the Netherlands (Brouwer-Borghuis et al., 2019) reported that at least one third of autistic adolescents in a school-based intervention for school refusal had experience of bullying, with a similar pattern among the other participants. A Japanese study (Ochi et al., 2020) reported significant association of bullying with school refusal in a retrospective chart review for autistic outpatients at a psychiatric hospital. A study in the United States of students diagnosed with autism without intellectual disability (Ashburner et al., 2019) found that those with co-occurring anxiety disorders and depression were more likely to report victimization, while parents reported that bullying had an impact on school attendance.

##### Individual factors

As mentioned above, some population-based studies (Fleming et al., 2020; John et al., 2022; Stromberg et al., 2022) indicated that co-occurring neurodevelopmental or mental conditions increased the risk substantially for school absenteeism in autistic children. A study from the United States (McClemont et al., 2021) noted that school refusal in children with co-occurring autism and ADHD was related to behavioral problems. A retrospective cohort study of students attending state secondary schools also found that poor attendance at school was associated with subsequent self-harm for children with and without autism (Widnall et al., 2022). The Australian study discussed above in relation to bullying found that autistic boys with emerging school refusal due to bullying experienced a significantly higher level of separation anxiety than their peers (Bitsika, Sharpley, & Heyne, 2022), and an inverse association between psychological resilience and emerging school refusal (Bitsika, Heyne, & Sharpley, 2022).

Individual characteristics of autistic children in mainstream classes were analyzed by parental questionnaires in a Norwegian report (Munkhaugen et al., 2019). In comparison to autistic children without school-attendance problems, those with school refusal behavior were significantly less socially motivated, displayed more deficits in initiating task or activities, were more withdrawn, and had more depressive symptoms. A Japanese study (Matsuura et al., 2020) found that children demonstrating school refusal behavior had significantly lower quality of life than a comparison group. However, this association could not be established for autistic children, possibly due to the low number of individuals with this diagnosis in the sample. A study from the United States, aiming to develop a machine learning algorithm to predict school absenteeism, failed to identify specific predictors. However, the authors suggest that attendance data for autistic children and signs of maladaptive behavior may be of help in early detection (Jarbou et al., 2022).

Co-existing intellectual disability was not consistently linked to school absenteeism; conversely, one study suggested that autistic children with school refusal behavior were cognitively more able than those without (Kurita, 1991). In a study of autistic children with co-occurring or suspected intellectual disability, a measure of adaptive skills was not significantly correlated with absence (Mattson et al., 2022).

Three studies reported that school absenteeism in autistic children became more common with age (Adams, 2022; John et al., 2022; Totsika et al., 2020). The population-based study by John et al. (2022) also reported that autistic girls were more likely to be absent than were boys, while the pattern was reversed for exclusion.

##### Family factors

Family factors were investigated as possible antecedents of school absenteeism in three studies, all mentioned above. The Australian online survey targeted to parents (Adams, 2022) found associations of parental unemployment and parental mental health with non-attendance. The online survey from the United Kingdom found that parental unemployment and not living in a two-parent household were associated with non-attendance (Totsika et al., 2020). Finally, an association between school refusal behavior and illness of family members was observed in the Norwegian study on autistic children in mainstream classes (Munkhaugen et al., 2019).

##### Evaluation of tools for assessment of contextual factors

An Australian study (Adams et al., 2022) evaluated an instrument originally developed for assessment of causes for school refusal in neurotypical pupils, the School Refusal Assessment Scale–Revised–Parent version (SRAS-R-P; Kearney, 2002). Feedback from parents suggested that items on autism-specific school-related factors were missing, including sensory environment, teacher–child relationships, and knowledge about autism among school staff.

### Interventions

Eleven studies with focus on support or treatment were identified. In this section, we included not only studies evaluating intervention programs but also case studies and qualitative studies tapping the experiences of students, clinicians, and school staff related to support or treatment (Table 3).

#### Summary of individual studies

##### Targeted interventions

Four studies described detailed intervention programs. The Link program from the Netherlands aims to link adolescents with school refusal back into education (Brouwer-Borghuis et al., 2019). Data from 30 participants were reported, of whom 18 were diagnosed with autism. No adolescent was re-engaged with the original school, but participants were able to take part in other forms of education or training. A feasibility study of an intensive, school-based program in the United States included 61 participants with developmental disorders (59% with autism), many of whom had co-occurring medical disorders and mental health problems (Lambros et al., 2016). The authors found that the number of absences and school suspensions decreased compared to the year before. A study from the United Kingdom described a program for students with autism, anxiety, and severe non-attendance (Preece & Howley, 2018). The authors reported positive effects on attendance and well-being for the five students taking part in the program for 1 year.

The interventions had some aspects in common. The importance of building relationships was emphasized—to teachers and peers, between staff and family, and collaboration with professionals outside school. An individualized and flexible approach with adaptation of the learning situation was also stressed, including providing “a safe place” for the student. Psychoeducation and behavioral strategies were used, and an accepting and open climate at school was found to be important. Special consideration was also given to bullying.

In a Swedish study on social skills group training in mainstream upper secondary schools, students, teachers, and school leaders perceived positive effects of the intervention on school attendance and social school climate (Leifler et al., 2022). Four additional case studies were identified, focusing on cognitive-behavioral therapy in a fictionalized case with autism, obsessive-compulsive disorder (OCD), and gender diversity (Guastello et al., 2023), behavioral intervention for an autistic child with chronic migraine (Arvans & LeBlanc, 2009), hypnosis as anxiety management (Byron, 2002), and pharmacological treatment for an autistic child with generalized anxiety disorder (Yamada et al., 2020).

##### General support and treatment

A study from the United Kingdom included semi-structured interview with three autistic girls with emotionally based school avoidance, who were successfully re-engaged in mainstream school (O’Hagan et al., 2022). Developing a trusting student–key adult relationship was reported to be the first phase of re-engagement. In a Swedish study based on interviews (Melin et al., 2022), clinicians in child and adolescent psychiatric setting reported that many patients with severe attendance problems at school had difficulties related to autism. The importance of collaboration between the school and the parents was stressed, and a change of school was often necessary. In a qualitative study from Japan (Hirata & Ozawa, 2023), school counselors reported that school refusal was a common reason for giving support to children with developmental disorders, including autism. The authors highlighted the importance of early detection and intervention.

## Discussion

This is the first scoping review on school absenteeism in autism. The major themes covered by the accumulated research were occurrence of different forms of school absenteeism, the relevance of contextual factors, and strategies to intervene. Large-scale population-based studies suggested that children on the autism spectrum have higher risk of school absenteeism compared to their non-autistic peers, with particularly high rates among those with co-occurring mental, behavioral, or neurodevelopmental conditions (Fleming et al., 2020; John et al., 2022; Stromberg et al., 2022). More severe disability and co-occurring health conditions were linked to authorized absence (Hatton, 2018; Lee et al., 2008; Totsika et al., 2020). Bullying also emerged as a possible risk factor, which may result in a downward spiral where children get fewer opportunities to practice social skills and become increasingly marginalized and anxious (Bitsika, Heyne, & Sharpley, 2022; Forrest et al., 2020; Wainscot et al., 2008). The few available studies on targeted interventions were based on few participants, and studies on early detection were scarce.

To better understand the need for further research in this field, it is important to consider school absenteeism in general and if there are aspects of this phenomenon that are specific to autism. Some of the large population-based studies included in our review examined a range of other conditions, allowing for comparison. The estimated risk among children with other neurodevelopmental conditions (e.g. ADHD and learning disabilities) were similar or somewhat higher than among autistic children, while the risk was substantially higher for children with common mental health conditions such as depression, bipolar disorder, and substance use disorders (Fleming et al., 2020; John et al., 2022). This aligns well with the adjusted estimates from the same studies, where the increased risk seen in autistic children at least partly was attributable to co-occurring conditions. Some factors causing and maintaining absenteeism may be quite generic (Leduc et al., 2022), while other factors may be related more directly to autism (e.g. high risk of bullying, insufficient accommodations, and a lack of knowledge about autism). Consequently, it is unclear if effect estimates for more generic interventions can be generalized to autistic children. Indeed, the quite modest and inconsistent intervention effects reported in recent meta-analyses (Eklund et al., 2022; Maynard et al., 2018) hint at the possibility that further adaptations may be necessary to the individual needs and preferences of individual students.

Research on school absenteeism in autistic children evidently has picked up speed in the last few years, but there are still some striking gaps. No studies from low- and middle-income countries were identified, despite widely reported difficulties for children with disabilities in primary and secondary school in these regions (World Bank Group, 2019). There is also a need for more research into the mechanisms leading to and maintaining school absenteeism in autistic children. The quite sizable number of studies on contextual factors point to the multifactorial nature of the phenomenon. Still, most factors were investigated in only one or a few studies. Importantly, few studies on sex differences were identified and study participants were predominantly male. Longitudinal studies were also lacking, precluding conclusions about temporality.

There is also a clear need for more robust studies on intervention effects. The overall complexity of school absenteeism in autistic children suggests that interventions may be needed at multiple levels, including school, individual, and family. A close collaboration with mental health services and social services seems crucial. The few available programs that were developed and preliminary evaluated will be a good starting point for future research, along with a recent practitioner review summarizing available treatment protocols (Heyne, 2022). In addition, the need for support and necessary accommodations in school should be further explored (L. Anderson, 2020).

Similarly, reliable tools for detection of early signs of school attendance problems are needed, as well as guidelines for determining when to intervene. Autism-specific information related to this is scant and proper adaptation of more generic tools seems crucial (Adams et al., 2022). For instance, a recently proposed framework based on functional impairment in important life domains (Kearney, 2022) could potentially be adapted to this specific population.

While the overall body of research focusing on school absenteeism and autism evidently is quite substantial, systematic reviews with a narrow focus on central review questions may still be premature. The varied and inconsistent use of terminology and definitions related to occurrence is a major challenge. On the positive side, though, ongoing initiatives to promote consensus on constructs and measurement methods in research (Heyne et al., 2020) have the potential to help facilitate future collection of data that will be comparable across different cultures and settings. A more detailed mapping of the contextual factors investigated, and the magnitude of the associations reported, could be a starting point for further disentanglement of mechanisms. However, further syntheses of the evidence may currently prove fruitless due to few studies focusing on each factor and heterogeneity across studies in terms of design, study population, and definitions. As for intervention effects, further synthesis will not be feasible due to the heterogeneous interventions, designs, and outcomes used in the few available studies.

### Limitations

The overarching goal of this scoping review was to provide an overview of the field to guide future research, not to support decision-making and implementation in practice. No review questions related to the results were pre-registered, and there was no critical appraisal of the included sources of evidence. Furthermore, we did not review gray literature (e.g. reports from government agencies or interest organizations). There is also a risk that we failed to identify some reports with relevant information, even though we obtained almost 600 reports in full text. This risk may be heightened by the inconsistent use of terminology in this field. Furthermore, studies focusing on diverse conditions and disabilities were excluded, unless data were reported separately for the autistic participants. This may have resulted in the exclusion of some relevant information.

## Conclusion

School absenteeism is a major challenge for autistic children and their families, often arising in a context of other developmental, health-related, and psychosocial problems. Further research is needed, especially on causal and maintaining factors, early detection, targeted intervention, and the situation in low- and middle-income countries. Pending further evidence, schools should make necessary accommodations to promote school attendance among children on the autism spectrum.

## Supplemental Material

sj-docx-1-aut-10.1177_13623613231217409 – Supplemental material for School absenteeism in autistic children and adolescents: A scoping review Supplemental material, sj-docx-1-aut-10.1177_13623613231217409 for School absenteeism in autistic children and adolescents: A scoping review by Viviann Nordin, Maud Palmgren, Anna Lindbladh, Sven Bölte and Ulf Jonsson in Autism

sj-docx-2-aut-10.1177_13623613231217409 – Supplemental material for School absenteeism in autistic children and adolescents: A scoping review Supplemental material, sj-docx-2-aut-10.1177_13623613231217409 for School absenteeism in autistic children and adolescents: A scoping review by Viviann Nordin, Maud Palmgren, Anna Lindbladh, Sven Bölte and Ulf Jonsson in Autism

sj-docx-3-aut-10.1177_13623613231217409 – Supplemental material for School absenteeism in autistic children and adolescents: A scoping review Supplemental material, sj-docx-3-aut-10.1177_13623613231217409 for School absenteeism in autistic children and adolescents: A scoping review by Viviann Nordin, Maud Palmgren, Anna Lindbladh, Sven Bölte and Ulf Jonsson in Autism

sj-docx-4-aut-10.1177_13623613231217409 – Supplemental material for School absenteeism in autistic children and adolescents: A scoping review Supplemental material, sj-docx-4-aut-10.1177_13623613231217409 for School absenteeism in autistic children and adolescents: A scoping review by Viviann Nordin, Maud Palmgren, Anna Lindbladh, Sven Bölte and Ulf Jonsson in Autism
