# Supplementary material for: School absenteeism in autistic children and adolescents: A scoping review
Source: Autism. 2023 Dec 30;28(7):1622–37. doi: 10.1177/13623613231217409 (PMC11191666; doi:10.1177/13623613231217409)
Supplement: sj-docx-1-aut-10.1177_13623613231217409 – Supplemental material for School absenteeism in autistic children and adolescents: A scoping review [file sj-docx-1-aut-10.1177_13623613231217409.docx]

Appendix A: Search strategies

School absenteeism in autistic children and adolescents – A scoping review

Contents

[Documentation of search strategies – University Library search consultation group 2](#_Toc134038076)

[Medline 3](#_Toc134038077)

[PsycInfo 5](#_Toc134038078)

[ERIC (ProQuest) 8](#_Toc134038079)

[Web of Science Core Collection 9](#_Toc134038080)

[Cochrane Library 10](#_Toc134038081)

# Documentation of search strategies – University Library search consultation group

Databases:

1. Medline (Ovid)
2. PsycInfo (Ebsco)
3. ERIC (ProQuest)
4. Web of Science Core Collection
5. Cochrane Library (Wiley)

Total number of hits:

- Before deduplication: 6,419
- After deduplication: 4,632

Search strategy

A literature search was performed in the following databases: Medline, PsycInfo, ERIC, Web of Science Core Collection, and Cochrane. After the original search was performed on 12 January 2017, the search was last updated on June 9, 2023 using the methods described by Bramer et al (1)

The search strategy was developed in Medline (Ovid) in collaboration with librarians. For each search concept Medical Subject Headings (MeSH-terms) and free text terms were identified. The search was then translated into the other databases. No language restriction was applied. Databases were searched from inception. The strategies were peer reviewed by another librarian prior to execution. De-duplication was done using the method described by Bramer et al (2).

References:

1. Bramer W, Bain P. (2017). Updating search strategies for systematic reviews using EndNote. *Journal of the Medical Library Association: JMLA*, 105(3):285-289. doi: 10.5195/jmla.2017.183.

2. Bramer, W. M., Giustini, D., de Jonge, G. B., Holland, L., & Bekhuis, T. (2016). De-duplication of database search results for systematic reviews in EndNote. *Journal of the Medical Library Association: JMLA*, 104(3), 240-243. doi: 10.3163/1536-5050.104.3.014

# Medline

| Interface: Ovid MEDLINE(R) and Epub Ahead of Print, In-Process & Other Non-Indexed Citations and Daily  Date of Search: 9 June 2023  Number of hits: 1315  Comment: In Ovid, two or more words are automatically searched as phrases; i.e. no quotation marks are needed | Field labels   - exp/ = exploded MeSH term - / = non exploded MeSH term - .ti,ab,kf. = title, abstract and author keywords - adjx = within x words, regardless of order - * = truncation of word for alternate endings |
| --- | --- |
| Database(s): **Ovid MEDLINE(R) ALL**1946 to June 08, 2023 Search Strategy:   \| **#** \| **Searches** \| **Results** \| \| --- \| --- \| --- \| \| 1 \| exp Neurodevelopmental disorders/ \| 208716 \| \| 2 \| Rett syndrome/ \| 2933 \| \| 3 \| ((attention deficit or behavio?r* or communication or conduct or fluency or intellectual* or hyperkinetic or hyperactiv* or learning or motor skill* or neurodevelopmental or neuropsychiatric* or reactive attachment or rett* or pervasive developmental or reading or speech sound or tic) adj3 (deficit* or disabilit* or disorder* or disease* or dysfunction* or impairment* or syndrome*)).ti,ab,kf. \| 190846 \| \| 4 \| (acalculi* or adhd or asperger* or asd or autis* or childhood schizophrenia or developmental disabilit* or dyscalculi* or dyslexi* or dyslectic or mental* retard* or mutism or pdd or separation anxiety or tourette*).ti,ab,kf. \| 161807 \| \| 5 \| or/1-4 \| 376534 \| \| 6 \| Absenteeism/ \| 9760 \| \| 7 \| Student dropouts/ \| 1889 \| \| 8 \| (absenteeism* or early school leav* or truan*).ti,ab,kf. \| 8069 \| \| 9 \| ((classroom* or school) adj1 (anxiety or avoid* or fear* or phobi* or refus* or reluctan*)).ti,ab,kf. \| 880 \| \| 10 \| ((class or classes or school) adj3 (miss* or skip*)).ti,ab,kf. \| 2030 \| \| 11 \| ((class or classes or classroom* or college* or education* or school* or student*) adj3 (absen* or attendance or dropout* or drop* out*)).ti,ab,kf. \| 9527 \| \| 12 \| ((college* or education* or school*) adj3 (cessation* or completion* or disrupt* or graduation or quit*)).ti,ab,kf. \| 4559 \| \| 13 \| ((out of school or non enrolled) adj1 (adolescen* or boy* or girl* or child* or youth or teenager*)).ti,ab,kf. \| 342 \| \| 14 \| or/6-13 \| 30174 \| \| 15 \| 5 and 14 \| 1325 \| \| 16 \| remove duplicates from 15 \| 1315 \| | |

PsycInfo

| Interface: Ebsco  Date of Search: 9 June 2023  Number of hits: 2,210  Comment: In Ovid, two or more words are automatically searched as phrases; i.e. no quotation marks are needed | Field labels   - DE = controlled term - TI,AB,KW = title, abstract and author keywords - Nx = within x words, regardless of order - * = truncation of word for alternate endings |
| --- | --- |
| \| **#** \| **Query** \| **Results** \| \| --- \| --- \| --- \| \| S30 \| S15 AND S29 \| 2,210 \| \| S29 \| S16 OR S17 OR S18 OR S19 OR S20 OR S21 OR S22 OR S23 OR S24 OR S25 OR S26 OR S27 OR S28 \| 32,090 \| \| S28 \| TI ( (("out of school" or "non enrolled") N1 (adolescen* or boy* or girl* or child* or youth or teenager*)) ) OR AB ( (("out of school" or "non enrolled") N1 (adolescen* or boy* or girl* or child* or youth or teenager*)) ) OR KW ( (("out of school" or "non enrolled") N1 (adolescen* or boy* or girl* or child* or youth or teenager*)) ) \| 388 \| \| S27 \| TI ( ((college* or education* or school*) N3 (cessation* or completion* or disrupt* or graduation or quit*)) ) OR AB ( ((college* or education* or school*) N3 (cessation* or completion* or disrupt* or graduation or quit*)) ) OR KW ( ((college* or education* or school*) N3 (cessation* or completion* or disrupt* or graduation or quit*)) ) \| 7,419 \| \| S26 \| TI ( ((class or classes or classroom* or college* or education* or school* or student*) N3 (absen* or attendance or dropout* or "drop* out*")) ) OR AB ( ((class or classes or classroom* or college* or education* or school* or student*) N3 (absen* or attendance or dropout* or "drop* out*")) ) OR KW ( ((class or classes or classroom* or college* or education* or school* or student*) N3 (absen* or attendance or dropout* or "drop* out*")) ) \| 14,209 \| \| S25 \| TI ( ((class or classes or school) N3 (miss* or skip*)) ) OR AB ( ((class or classes or school) N3 (miss* or skip*)) ) OR KW ( ((class or classes or school) N3 (miss* or skip*)) ) \| 2,816 \| \| S24 \| TI ( ((classroom* or school) N1 (anxiety or avoid* or fear* or phobi* or refus* or reluctan*)) ) OR AB ( ((classroom* or school) N1 (anxiety or avoid* or fear* or phobi* or refus* or reluctan*)) ) OR KW ( ((classroom* or school) N1 (anxiety or avoid* or fear* or phobi* or refus* or reluctan*)) ) \| 2,603 \| \| S23 \| TI ( (absenteeism* or "early school leav*" or truan*) ) OR AB ( (absenteeism* or "early school leav*" or truan*) ) OR KW ( (absenteeism* or "early school leav*" or truan*) ) \| 6,548 \| \| S22 \| DE School Phobia \| 376 \| \| S21 \| DE School Graduation \| 989 \| \| S20 \| DE Student Attrition \| 565 \| \| S19 \| DE School Truancy \| 604 \| \| S18 \| DE School Refusal \| 470 \| \| S17 \| DE "School Dropouts" OR DE "College Dropouts" \| 1 \| \| S16 \| DE School Attendance \| 2,652 \| \| S15 \| S1 OR S2 OR S3 OR S4 OR S5 OR S6 OR S7 OR S8 OR S9 OR S10 OR S11 OR S12 OR S13 OR S14 \| 309,085 \| \| S14 \| TI ( (acalculi* OR adhd OR asperger* OR asd OR autis* OR "childhood schizophrenia" OR "developmental disabilit*" OR dyscalculi* OR dyslexi* OR dyslectic OR "mental* retard*" OR mutism OR pdd OR "separation anxiety" OR tourette*) ) OR AB ( (acalculi* OR adhd OR asperger* OR asd OR autis* OR "childhood schizophrenia" OR "developmental disabilit*" OR dyscalculi* OR dyslexi* OR dyslectic OR "mental* retard*" OR mutism OR pdd OR "separation anxiety" OR tourette*) ) OR KW ( (acalculi* OR adhd OR asperger* OR asd OR autis* OR "childhood schizophrenia" OR "developmental disabilit*" OR dyscalculi* OR dyslexi* OR dyslectic OR "mental* retard*" OR mutism OR pdd OR "separation anxiety" OR tourette*) ) \| 160,782 \| \| S13 \| TI ( (("attention deficit" OR behavio#r* OR communication OR conduct OR fluency OR intellectual* OR hyperkinetic OR hyperactiv* OR learning OR "motor skill*" OR neurodevelopmental OR neuropsychiatric* OR "reactive attachment" OR rett* OR "pervasive developmental" OR reading OR "speech sound" OR tic) N3 (deficit* OR disabilit* OR disorder* OR disease* OR dysfunction* OR impairment* OR syndrome*)) ) OR AB ( (("attention deficit" OR behavio#r* OR communication OR conduct OR fluency OR intellectual* OR hyperkinetic OR hyperactiv* OR learning OR "motor skill*" OR neurodevelopmental OR neuropsychiatric* OR "reactive attachment" OR rett* OR "pervasive developmental" OR reading OR "speech sound" OR tic) N3 (deficit* OR disabilit* OR disorder* OR disease* OR dysfunction* OR impairment* OR syndrome*)) ) OR KW ( (("attention deficit" OR behavio#r* OR communication OR conduct OR fluency OR intellectual* OR hyperkinetic OR hyperactiv* OR learning OR "motor skill*" OR neurodevelopmental OR neuropsychiatric* OR "reactive attachment" OR rett* OR "pervasive developmental" OR reading OR "speech sound" OR tic) N3 (deficit* OR disabilit* OR disorder* OR disease* OR dysfunction* OR impairment* OR syndrome*)) ) \| 171,219 \| \| S12 \| DE Rett Syndrome \| 1,072 \| \| S11 \| DE "Childhood Schizophrenia" \| 0 \| \| S10 \| DE "Attachment Disorders" OR DE "Disinhibited Social Engagement Disorder" \| 835 \| \| S9 \| DE "Mutism" OR DE "Elective Mutism" \| 749 \| \| S8 \| DE "Tourette Syndrome" \| 3,701 \| \| S7 \| DE Tics \| 2,144 \| \| S6 \| DE "Developmental Disabilities" OR DE "Specific Language Impairment" \| 20,588 \| \| S5 \| DE "Communication Disorders" \| 3,389 \| \| S4 \| DE "Acalculia" \| 0 \| \| S3 \| DE "Behavior Disorders" \| 9,786 \| \| S2 \| DE "Separation Anxiety" OR DE "Separation Anxiety Disorder" \| 1,726 \| \| S1 \| DE "Neurodevelopmental Disorders" OR DE "Attention Deficit Disorder" OR DE "Autism Spectrum Disorders" OR DE "Developmental Disabilities" OR DE "Disruptive Behavior Disorders" OR DE "Emotional and Behavioral Disorders" OR DE "Intellectual Development Disorder" OR DE "Learning Disorders" OR DE "Attention Deficit Disorder with Hyperactivity" OR DE "Autistic Traits" OR DE "Specific Language Impairment" OR DE "Conduct Disorder" OR DE "Oppositional Defiant Disorder" OR DE "Anencephaly" OR DE "Crying Cat Syndrome" OR DE "Down's Syndrome" OR DE "Tay Sachs Disease" OR DE "Learning Disabilities" OR DE "Dyslexia" OR DE "Reading Disabilities" \| 191,836 \| | |

ERIC (ProQuest)

| Interface: ProQuest  Date of Search: 9 June 2023  Number of hits: 1,285 | Field labels   - MAINSUBJECT.EXACT.EXPLODE = exploded subject heading - MAINSUBJECT.EXACT non exploded subject heading - TI,AB = title, abstract - N/x = within x words, regardless of order - * = truncation of word for alternate endings |
| --- | --- |
| (MAINSUBJECT.EXACT.EXPLODE("Attendance" OR "Dropout Characteristics" OR "Dropout Prevention" OR "Dropout Programs" OR "Dropout Research" OR "Dropout Attitudes") OR MAINSUBJECT.EXACT("Dropouts" OR "Truancy" OR "Potential Dropouts" OR "Attendance Patterns" OR "Student Attrition" OR "Graduation" OR "School Phobia") OR TI,AB(absenteeism OR "early school leav*" OR truant OR truancy) OR TI,AB((classroom OR classrooms OR school) N/1 (anxiety OR avoid OR avoiding OR fear OR fears OR phobia OR phobias OR phobic OR refuse OR refusing OR reluctant or reluctance)) OR TI,AB((class OR classes OR school) N/3 (miss* OR skip*)) OR TI,AB((class OR classes OR classroom OR classrooms OR college OR colleges OR education OR educations OR student OR students) N/3 (absen* OR attendance OR dropout OR dropouts OR "drop* out*")) OR TI,AB((college OR colleges OR education OR educations OR school*) N/3 (cessation OR cessations OR completion OR completions OR disrupt* OR graduation OR quit*)) OR TI,AB(("out of school" OR "non enrolled") N/1 (adolescen* OR boy OR boys OR girl OR girls OR child OR children OR childs OR youth OR teenager OR teenagers)))  AND  (MAINSUBJECT.EXACT.EXPLODE("Neurodevelopmental Disorders" OR "Behavior Disorders") OR MAINSUBJECT.EXACT("Separation Anxiety" OR "Communication Disorders" OR "Dyslexia") OR TI,AB(("attention deficit" OR behavior OR behaviors OR behaviour OR behaviours OR communication OR conduct OR fluency OR intellectual* OR hyperkinetic OR hyperactive OR hyperactivity OR learning OR "motor skill" OR "motor skills" OR developmental OR neuropsychiatric* OR "reactive attachment" OR rett* OR "pervasive developmental" OR reading OR "speech sound" OR tic) N/3 (deficit* OR disabilit* OR disorder OR disorders OR disease OR diseases OR dysfunction OR dysfunctional OR impairment OR impairments OR syndrome OR syndromes)) OR TI,AB(acalculi* OR add OR asperger* OR ash OR autism OT autistic OR "childhood schizophrenia" OR "developmental disabilities" OR "developmental disability" OR dyscalculi* OR dyslexi* OR dyslexic OR "mental* retard* " OR mutism OR pdq OR "separation anxiety" OR tourette OR tourettes)) | |

Web of Science Core Collection

| Interface: Clarivate Analytics  Editions = A&HCI , ESCI , SCI-EXPANDED , SSCI  Date of Search: 9 Juni 2023  Number of hits: 1,354 | Field labels   - TS/Topic = title, abstract, author keywords and Keywords Plus - NEAR/x = within x words, regardless of order - * = truncation of word for alternate endings   Note: the *Exact search*-function was used for all the searches |
| --- | --- |
| \| **#** \| **Search Query** \| **Results** \| \| --- \| --- \| --- \| \| 1 \| TS=(("attention deficit" OR behavior* OR behaviour* OR communication OR conduct OR fluency OR intellectual* OR hyperkinetic OR hyperactiv* OR learning OR "motor skill*" OR neurodevelopmental OR neuropsychiatric* OR "reactive attachment" OR rett* OR "pervasive developmental" OR reading OR "speech sound" OR tic) NEAR/3 (deficit* OR disabilit* OR disorder* OR disease* OR dysfunction* OR impairment* OR syndrome*) ) \| 263163 \| \| 2 \| TS=(acalculi* OR adhd OR asperger* OR asd OR autis* OR "childhood schizophrenia" OR "developmental disabilit*" OR dyscalculi* OR dyslexi* OR dyslectic OR "mental* retard*" OR mutism OR pdd OR "separation anxiety" OR tourette*) \| 223368 \| \| 3 \| #2 OR #1 \| 406772 \| \| 4 \| TS=(absenteeism* OR "early school leav*" OR truan*) \| 10930 \| \| 5 \| TS=((classroom* OR school) NEAR/1 (anxiety OR avoid* OR fear* OR phobi* OR refus* OR reluctan*) ) \| 2143 \| \| 6 \| TS=((class OR classes OR school) NEAR/3 (miss* OR skip*) ) \| 3503 \| \| 7 \| TS=((class OR classes OR classroom* OR college* OR education* OR school* OR student*) NEAR/3 (absen* OR attendance OR dropout* OR "drop* out*") ) \| 17554 \| \| 8 \| TS=((college* OR education* OR school*) NEAR/3 (cessation* OR completion* OR disrupt* OR graduation OR quit*) ) \| 8824 \| \| 9 \| TS=(("out of school" OR "non enrolled") NEAR/1 (adolescen* OR boy* OR girl* OR child* OR youth OR teenager*) ) \| 537 \| \| 10 \| #9 OR #8 OR #7 OR #6 OR #5 OR #4 \| 39608 \| \| 11 \| #10 AND #3 \| 1354 \| | |

Cochrane Library

| Interface: Wiley  Date of Search: 9 Juni 2023  Number of hits: 255   - Cochrane Reviews = 6 - Trials = 249 | Field labels   - ti,ab,kw = title, abstract and author keywords - NEAR/x = within x words, regardless of order - * = truncation of word for alternate endings |
| --- | --- |
| \| ID \| Search \| Hits \| \| --- \| --- \| --- \| \| #1 \| (("attention deficit" or behavior* or behaviour* or "communication" or "conduct" or "fluency" or intellectual* or "hyperkinetic" or hyperactiv* or "learning" or "motor skill" or "motor skills" or "neurodevelopmental" or neuropsychiatric* or "reactive attachment" or rett* or "pervasive developmental" or "reading" or "speech sound" or "tic") NEAR/3 (deficit* or disabilit* or disorder* or disease* or dysfunction* or impairment* or syndrome*)):ti,ab,kw \| 24226 \| \| #2 \| (acalculi* or "adhd" or asperger* or "asd" or autis* or "childhood schizophrenia" or "developmental disability" or "developmental disabilities" or dyscalculi* or dyslexi* or "dyslectic" or (mental* NEXT retard*) or "mutism" or "pdd" or "separation anxiety" or tourette*):ti,ab,kw \| 14811 \| \| #3 \| #1 or #2 \| 31432 \| \| #4 \| (absenteeism* or (early NEXT school NEXT leav*) or truan*):ti,ab,kw \| 2164 \| \| #5 \| (classroom* or "school") NEAR/1 ("anxiety" or avoid* or fear* or phobi* or refus* or reluctan*):ti,ab,kw \| 87 \| \| #6 \| ("class" or "classes" or "school") NEAR/3 (miss* or skip*):ti,ab,kw \| 335 \| \| #7 \| ("class" or "classes" or classroom* or college* or education* or school* or student*) NEAR/3 (absen* or "attendance" or dropout* or "drop* out*"):ti,ab,kw \| 1819 \| \| #8 \| (college* or education* or school*) NEAR/3 (cessation* or completion* or disrupt* or graduation or quit*):ti,ab,kw \| 664 \| \| #9 \| ("out of school" or "non enrolled") NEAR/1 (adolescen* or boy* or girl* or child* or "youth" or teenager*):ti,ab,kw \| 25 \| \| #10 \| #4 or #5 or #6 or #7 or #8 or #9 \| 4589 \| \| #11 \| #3 AND #10 \| 255 \| | |
