# Supplementary material for: School absenteeism in autistic children and adolescents: A scoping review
Source: Autism. 2023 Dec 30;28(7):1622–37. doi: 10.1177/13623613231217409 (PMC11191666; doi:10.1177/13623613231217409)
Supplement: sj-docx-2-aut-10.1177_13623613231217409 – Supplemental material for School absenteeism in autistic children and adolescents: A scoping review [file sj-docx-2-aut-10.1177_13623613231217409.docx]

Appendix C: Supplemental tables

School absenteeism in autistic children and adolescents – A scoping review

Contents

[**Table S1.** Definitions of school absenteeism in studies focusing on occurrence. 2](#_Toc142146068)

[**Table S2.** Definitions of school absenteeism in studies focusing on context. 5](#_Toc142146069)

[**Table S3.** Definitions of school absenteeism in studies on interventions. 9](#_Toc142146070)

## **Table S1.** Definitions of school absenteeism in studies focusing on occurrence.

| **Author**  **(Publication year)**  **Country** | **Main form of**  **absence** | **Subtype(s) of**  **absence** | **Measurement of**  **absence** | **Thresholds** |
| --- | --- | --- | --- | --- |
| Adams  (2022)  Australia | School non-attendance | “Non-problematic” absence, school refusal, and other types from the School Non-Attendance ChecKlist, SNACK (Heyne et al 2019). | Number of full-day and half-day absences in 4 weeks (20-days). | Persistent absence defined as 3 days or more in 20 days |
| Anderson  (2021)  USA | Absenteeism  Exclusionary discipline | Absenteeism: ---  Exclusions: in-school and out-of-school suspension, expulsion or removal to alternative education | Percent of days absent in a year | Chronically absent: defined as missing at least 10% of school days in a year |
| Chen et al.  (2016)  USA | Truancy | Unexcused/excused  school absence | Days/month, every month during a school year. | Classified in five trajectory subgroups (very low, low, declining, rising, chronic) |
| Crump et al.  (2013)  USA | School absenteeism | Regardless of reason | Number of full-day absences/year. | N/A |
| Fleming et al.  (2020)  UK | School absenteeism  Exclusion | Absenteeism: N/A  Exclusion: expulsions, suspensions | Annual number of days absent  Annual number of exclusions | N/A |
| Hatton et al.  (2018)  UK | School absences  School exclusions | Authorized/unauthorized absence  Fixed-period/permanent exclusions | Average percentage of half-day sessions throughout the year.  Average number of exclusions. | Persistent absentees: missed 10% or more of school sessions  One or more exclusion |
| John et al.  (2022)  UK | School absence (absenteeism)  Exclusion | Authorized and unauthorized absence  Any type of exclusion (lunchtime, fixed, permanent) | Number of absences per year  Number of exclusions per year | Absence: more than 10% of sessions in one year  Exclusion: at least once in one year |
| Kurita  (1991)  Japan | School refusal | Modified criteria of Berg et al (1969): absence without physical disease, with parental knowledge, no antisocial disorder | Having a history of school refusal on a 3-point scale (have had, shown unwillingness, never had) | School refusal or no school refusal |
| Lee et al.  (2008)  USA | Missing school | Absence because of illness or injury | Number of days missed school in 12 months | Missed more than one week of school (8 days or more) |
| Melvin et al (2023)  Australia | School absenteeism | School Attendance Problems (SAP) from the School Non-Attendance ChecKlist, SNACK (Heyne et al 2019): school refusal, school withdrawal, school exclusion and truancy. | Days or half-days absent in past 20 school days | N/A |
| Munkhaugen et al.  (2017)  Norway | School refusal behavior | Refusal behaviors divided in 2 categories: verbal/physical refusal behaviors and partial/complete unauthorized absence | Number of days with refusal behavior during 20 consecutive days | Any school refusal behaviors; yes or no. Absence divided in  1-3 days, 4-10 days, 11-20 days |
| Stromberg et al.  (2022)  USA | School absenteeism | Missed days because of illness or injury | Number of school days missed in past 12 months | Frequent/chronic absenteeism defined as more than 11 days |
| Takara & Kondo  (2014)  Japan | School non-attendance | N/A | Past history of school non-attendance (not specified). Data from 2 earlier studies by Kurita (1991) on school refusal. | N/A |
| Tani et al.  (2012)  Japan | School non-attendance | N/A | Life history with school non-attendance (not specified).  Data from interviews and (if available) school records | N/A |
| Totsika et al.  (2020)  UK | School non-attendance | Including school refusal, exclusion, withdrawal and truancy.  Non-problematic: related to illness. | Number of school-days missed in one month (23 school days) | Persistent non-attendance: more than 10% (3 days) |

N/A: Not applicable.

## **Table S2.** Definitions of school absenteeism in studies focusing on context.

| **Author**  **(Publication year)**  **Country** | **Main form of**  **absence** | **Subtype(s) of**  **absence** | **Measurement of**  **absence** | **Definitions/thresholds** |
| --- | --- | --- | --- | --- |
| Adams  (2022)  Australia | School non-attendance | “Non-problematic” absence, school refusal, and other types from the School Non-Attendance ChecKlist, SNACK (Heyne et al 2019). | Number of full-day and half-day absences in 4 weeks (20-days). | Persistent absence defined as 3 days or more in 20 days |
| Adams et al.  (2022)  Australia | School absence, absenteeism | Due to illness  Without illness | N/A | Short absences vs continuous periods of over 4 weeks |
| Anderson  (2020)  Sweden | School absence,  absenteeism | Due to illness  Without illness | N/A | “Short absences” vs  continuous periods more  than 4 weeks |
| Ashburner et al.  (2019)  USA | Reduced willingness to attend school | Negative impact of bullying on school attendance | Number/percentage of parents confirming bullying having impacted their child’s school attendance | N/A |
| Bitsika et al.  (2021)  Australia | School refusal | Emerging School Refusal (ESR) (concept by Ingul et al 2019) related to bullying and to separation anxiety | Number/percentage of boys exhibiting ESR due to bullying | N/A |
| Bitsika, Heyne et al.  (2022)  Australia | School refusal | Emerging School refusal (ESR) related to bullying and to psychological resilience | Number/percentage of boys exhibiting ESR due to bullying | N/A |
| Bitsika, Sharpley et al.  (2022)  Australia | School refusal | Emerging School refusal (ESR) related to bullying and to psychological resilience | Number/percentage of boys exhibiting ESR due to bullying | N/A |
| Brouwer-Borghuis et al.  (2019)  The Netherlands | School refusal (SR) | SR defined by Berg 1997. Related to school factors. | History of attendance in school records | Complete absence 5–87 weeks was defined as chronic |
|  |  |  |  |  |
| Feldman et al.  (2015)  USA | Presence during classes | Six profiles of presence pattern during classes | Percentage present/gone in the classroom during 3 classes | N/A |
| Fleming et al.  (2020)  UK | School absenteeism  Exclusion | Absenteeism: days absent  Exclusion: expulsions, suspensions | Annual number of days absent  Annual number of exclusions | N/A |
| Foster & Pearson  (2012)  USA | Drop out of high school | N/A | Proportion of time in general/special education, and association with not dropping out of high school | Coded as not dropping out if they graduated, received a GED certificate or were still in high school at wave 4 data collection. |
| Jarbou et al.  (2022)  USA | School absenteeism | Short- and long-term absenteeism | Students’ attendance history (3 or 12 months) used to predict future absenteeism. | Chronic absenteeism: missing more than 10% of annual school days |
| John et al.  (2022)  UK | School absence (absenteeism)  Exclusion | Authorized and unauthorized absence  Any type of exclusion (lunchtime, fixed, permanent) | Number of absences per year  Number of exclusions per year | Absence: more than 10% of sessions in one year  Exclusion: at least once in one year |
| Kouroupa  (2023)  UK | School absence | School refusal after COVID-19 lockdown, for children who had home-, school- and hybrid learning. | Mean number of school days absent in one month, after schools re-opening | Persistent absence defined as 10% or more of sessions |
| Kurita  (1991)  Japan | School refusal | Modified criteria of Berg et al (1969): absence without physical disease, with parental knowledge, no antisocial disorder | Having a history of school refusal on a 3-point scale (have had, shown unwillingness, never had) | School refusal or no school refusal |
| Lissack & Boyle  (2022)  UK | School non-attendance | N/A | Percentage of children experiencing attendance difficulties in 5 age-categories and 5 duration-categories | Age: 5-7, 8-10, 11-13, 14-16, 16+  Duration: <3m, 3-6m, 7-12m, 1-2y, >2y  Percentage with duration >2 years pointed out |
| Matsuura et al.  (2020)  Japan | School refusal behavior (SRB) | Four aspects of SRB assessed with the School Refusal Assessment Scale-Revised JA (Kearney 2002) | Number of days of absence per year | Persistent SRB: absent from school more than 30 days/year |
| Mattson et al.  (2022)  USA | School absenteeism | Excused (approved) and unexcused absence | Average number/median percent of days absent or tardy per year | Chronic absence defined as 10% or more in a school year (by US Department of Education) |
| McClemont et al.  (2021)  USA | School refusal | School refusal due to bullying | Parent reported occurrence of school refusal on a 4-point scale: never, in the past, this year, every month | N/A |
| Munkhaugen et al.  (2019)  Norway | School refusal behavior (SRB) | Including 6 levels of refusal behaviors and unexcused absenteeism (Kearney & Albano 2004) | Number of days of verbal/physical refusal or partial/complete absenteeism in 20 days | 3 frequency categories: 1-3 days, 4-10 days, 11-20 days of SRB, in 20 days |
| Ochi et al.  (2020)  Japan | School refusal (SR) | Unexcused SR according to the Japanese Ministry of Education (MEXT) | Data from psychiatric records, MEXT definition of SR (>30days) was inclusion criterion | MEXT defines school refusal as absence  > 30 days/year for reasons other than sickness or economic causes. |
| Paulauskaite  (2022)  UK | School de-registration and Elective Home Education (EHE) during COVID 19 | Pre-pandemic EHE and Pandemic EHE groups | Time and reasons for school de-registration, experiences of EHE | N/A |
| Totsika et al.  (2020)  UK | School non-attendance | Including school refusal, exclusion, withdrawal and truancy.  Non-problematic: related to illness. | Number of school-days missed in one month (23 school days) | Persistent non-attendance: more than 10% (3 days) |
| Wainscot et al.  (2008)  UK | School attendance | N/A | Mean attendance during past 50 days | N/A |
| Vincent  (2023)  UK | School lockdown during COVID-19 | N/A | The school experiences of autistic children and their parents before, during and after the lockdown | N/A |
| Widnall et al.  (2022)  UK | School absence  Exclusion | Self-harm and school absence related to ASD | Number/percentage of children with >80% attendance, or exclusions | Poor attendance: > 80% in one year  Record of fixed term exclusions |

N/A: Not applicable.

## **Table S3.** Definitions of school absenteeism in studies on interventions.

| **Author**  **(Year)**  **Country** | **Main form of**  **absence** | **Subtype(s) of**  **absence** | **Measurement of**  **absence** | **Thresholds** |
| --- | --- | --- | --- | --- |
| Arvans & LeBlanc  (2009)  USA | School absences | Absence related to migraine | Number of school days missed or shortened /week in a year | N/R |
| Brouwer-Borghuis et al.  (2019)  The Netherlands | School refusal (SR) | SR defined by Berg 1997. Related to school factors. | History of attendance in school records | Complete absence 5–87 weeks was defined as chronic |
| Byron  (2002)  UK | School attendance | Related to anxiety | N/A | N/A |
| Guastello et al.  (2023)  USA | School refusal | Related to anxiety and OCD | N/A | N/A |
| Hirata & Ozawa (2023) Japan | School refusal | Including school refusal, school refusal tendency, poor attendance | “Main complaint” reported by school counselors | N/A |
| Lambros et al.  (2016)  USA | School absences  Suspensions | Total number of absences  In-school and out-of-school suspensions | Number of absences (more than a half day) and number of suspensions, in a year prior to, and during the program | N/A |
| Leifler et al.  (2022)  Sweden | School attendance | N/A | Students and teachers perceived effects of the Social Skills Group Training SKOLKONTAKT on school attendance | N/A |
| Melin et al.  (2022)  Sweden | School attendance problems (SAP) | SAP associated with psychiatric comorbidity | N/A | Prolonged SAP: ranging from 6 moths to 3 years |
| O´Hagan et al.  (2022)  UK | School avoidance | Emotionally (anxiety) based school avoidance | Autistic girls’ experiences of supportive factors helping re-engagement in school | N/A |
| Preece & Howley  (2018)  UK | School refusal, school non-attendance | Absence associated with anxiety | Individual experiences and attendance records | N/A |
| Yamada et al.  (2020)  Japan | School refusal | Absence related to GAD | N/A | N/A |

N/A: Not applicable.
