# Supplementary material for: School absenteeism in autistic children and adolescents: A scoping review
Source: Autism. 2023 Dec 30;28(7):1622–37. doi: 10.1177/13623613231217409 (PMC11191666; doi:10.1177/13623613231217409)
Supplement: sj-docx-3-aut-10.1177_13623613231217409 – Supplemental material for School absenteeism in autistic children and adolescents: A scoping review [file sj-docx-3-aut-10.1177_13623613231217409.docx]

Appendix B: Excluded studies

School absenteeism in autistic children and adolescents – A scoping review

Contents

[Population 2](#_Toc135561689)

[No data on absenteeism in autistic children and adolescents 25](#_Toc135561690)

[Type of publication 38](#_Toc135561691)

[Not retrieved 48](#_Toc135561692)

## Population

Aguiar, A. L., Aguiar, C., Cadima, J., Correia, N., & Fialho, M. (2019). Classroom quality and children's social skills and problem behaviors: Dosage and disability status as moderators. *Early Childhood Research Quarterly, 49*, 81-92.

Akpan, M. U., Ojinnaka, N. C., & Ekanem, E. E. (2010). Academic performance of school children with behavioural disorders in Uyo, Nigeria. *Afr Health Sci, 10*(2), 154-158.

Anderson, J. A., Kutash, K., & Duchnowski, A. J. (2001). A comparison of the academic progress of students with EBD and students with LD. *Journal of Emotional and Behavioral Disorders, 9*(2), 106-115.

Andrews, C., Kakooza-Mwesige, A., Almeida, R., Swartling Peterson, S., Wabwire-Mangen, F., Eliasson, A. C., & Forssberg, H. (2019). Impairments, functional limitations, and access to services and education for children with cerebral palsy in Uganda: a population-based study. *Dev Med Child Neurol, 25*, 25.

Antshel, K. M., Faraone, S. V., & Gordon, M. (2014). Cognitive behavioral treatment outcomes in adolescent ADHD. *J Atten Disord, 18*(6), 483-495.

Askeland, K. G., Bøe, T., Sivertsen, B., Linton, S. J., Heradstveit, O., Nilsen, S. A., & Hysing, M. (2022). Association of depressive symptoms in late adolescence and school dropout. *School Mental Health: A Multidisciplinary Research and Practice Journal*.

Baeyens, D. (2021). Effectiveness of reasonable accommodations in students with ADHD: an experimental and intervention study. *Pedagogische Studien, 98*(4), 268-284.

Bain, A., & MacPherson, A. (1990). An Examination of the System-Wide Use of Exclusion with Disruptive Students. *Australia and New Zealand Journal of Developmental Disabilities, 16*(2), 109-123.

Ball, C., & Connolly, J. (2000). Educationally disaffected young offenders - Youth court and agency responses to truancy and school exclusion. *British Journal of Criminology, 40*(4), 594-616.

Barbaresi, W. J., Katusic, S. K., Colligan, R. C., Weaver, A. L., & Jacobsen, S. J. (2007). Long-term school outcomes for children with attention-deficit/hyperactivity disorder: a population-based perspective. *J Dev Behav Pediatr, 28*(4), 265-273.

Barbaresi, W. J., Katusic, S. K., Colligan, R. C., Weaver, A. L., & Jacobsen, S. J. (2007). Modifiers of long-term school outcomes for children with attention deficit/hyperactivity disorder: Does treatment with stimulant medication make a difference? Results from a population-based study. *Journal of Developmental and Behavioral Pediatrics, 28*(4), 274-287.

Barnett, W. S. (1998). Long-term cognitive and academic effects of early childhood education of children in poverty. *Preventive Medicine: An International Journal Devoted to Practice and Theory, 27*(2), 204-207.

Barwick, M. A., & Siegel, L. S. (1996). Learning difficulties in adolescent clients of a shelter for runaway and homeless street youths. *Journal of Research on Adolescence, 6*(4), 649-670.

Basch, C. E. (2011). Inattention and hyperactivity and the achievement gap among urban minority youth. *J Sch Health, 81*(10), 641-649.

Bear, G. G., Kortering, L. J., & Braziel, P. (2006). School completers and noncompleters with learning disabilities - Similarities in academic achievement and perceptions of self and teachers. *Remedial and Special Education, 27*(5), 293-300.

Beare, P. (1981). Mainstreaming approach for behaviorally disordered secondary students in a rural school district. *Behavioral Disorders, 6*(4), 209-218.

Beare, P. L. (1980). The In School-In Class Program: A Child Advocacy/Crisis Teacher Program for Behaviorally Disordered Students in a Rural School District. *Topical Conference of the Council for Exceptional Children*.

Benarous, X., Guedj, M. J., Cravero, C., Jakubowicz, B., Brunelle, J., Suzuki, K., & Cohen, D. (2022). Examining the hikikomori syndrome in a French sample of hospitalized adolescents with severe social withdrawal and school refusal behavior. *Transcultural Psychiatry*, 13634615221111633.

Bethell, C., Forrest, C. B., Stumbo, S., Gombojav, N., Carle, A., & Irwin, C. E. (2012). Factors promoting or potentially impeding school success: disparities and state variations for children with special health care needs. *Matern Child Health J, 16 Suppl 1*(Suppl 1), S35-43.

Bhakta, P., Hackett, R. J., & Hackett, L. (2002). The prevalence and associations of reading difficulties in a population of South Indian children. *Journal of Research in Reading, 25*(2), 191-202.

Biswas, H., & Sahoo, M. K. (2023). A study on psychiatric conditions in children with school refusal- A clinic based study. *Journal of Family Medicine & Primary Care, 12*(1), 160-164.

Bools, C., Foster, J., Brown, I., & Berg, I. (1990). The identification of psychiatric disorders in children who fail to attend school: a cluster analysis of a non-clinical population. *Psychol Med, 20*(1), 171-181.

Boras, S., & Zuckerman, Z. I. (2008). Influence of the Monitored Youth Mentoring Program for adolescents with behavioural problems and behavioural disorders. *Coll Antropol, 32*(3), 793-806.

Bouck, E. C., Bartz, K., & Costello, M. P. (2021). Transition Planning Involvement and Students with Intellectual Disability: Findings from the NLTS 2012. *Education and Training in Autism and Developmental Disabilities, 56*(2), 173-189.

Bowen, R., Chavira, D. A., Bailey, K., Stein, M. T., & Stein, M. B. (2008). Nature of anxiety comorbid with attention deficit hyperactivity disorder in children from a pediatric primary care setting. *Psychiatry Res, 157*(1-3), 201-209.

Bowman, L. J. (2004). Early Identification of Middle School At-Risk Students with Learning and Behavioral Disorders. *Multiple Voices for Ethnically Diverse Exceptional Learners, 7*(2), 48-59.

Boyle, C. A., Decoufle, P., & Yeargin-Allsopp, M. (1994). Prevalence and health impact of developmental disabilities in US children. *Pediatrics, 93*(3), 399-403.

Brill, C. L. (1994). The Effects of Participation in Service-Learning on Adolescents with Disabilities. *Journal of Adolescence, 17*(4), 369-380.

Brunstein-Klomek, A., Kopelman-Rubin, D., Apter, A., Argintaru, H., & Mufson, L. (2017). A pilot feasibility study of interpersonal psychotherapy in adolescents diagnosed with specific learning disorders, attention deficit hyperactive disorder, or both with depression and/or anxiety symptoms (IPT-ALD). *Journal of Psychotherapy Integration, 27*(4), 526-539.

Buchanan, P. S., & Scobie, R. P. (1988). Confrontation and Adaptation. *Academic Therapy, 23*(3), 315-322.

Buergi, B. R., Baloch, M. A., & Mengal, A. A. (2018). School-less or Out-of-school? Re-thinking Special Needs Education and Practice in Baluchistan, Pakistan. *International Journal of Special Education, 33*(2), 248-263.

Cairns, R. B., Cairns, B. D., & Neckerman, H. J. (1989). Early school dropout: configurations and determinants. *Child Dev, 60*(6), 1437-1452.

Canto, A. I., Proctor, B. E., & Prevatt, F. (2005). Educational Outcomes of Students First Diagnosed with Learning Disabilities in Postsecondary School. *Journal of College Admission, 187*, 8-13.

Carlberg, L., & Granlund, M. (2019). Achievement and participation in schools for young adolescents with self-reported neuropsychiatric disabilities: A cross-sectional study from the southern part of Sweden. *Scand J Public Health, 47*(2), 199-206.

Carter-Pokras, O. D., Bugbee, B. A., Gold, R. S., Lauver, P. E., Aiken, R., & Arria, A. M. (2019). Utilizing Student Health and Academic Data: A County-Level Demonstration Project. *Health Promot Pract*, 1524839919862796.

Cavendish, W. (2014). Academic Attainment During Commitment and Postrelease Education-Related Outcomes of Juvenile Justice-Involved Youth With and Without Disabilities. *Journal of Emotional and Behavioral Disorders, 22*(1), 41-52.

Cavendish, W., Connor, D. J., Olander, L., & Hallaran, A. (2020). Preparing for their Future: Perspectives of High School Students with Learning Disabilities about Transition Planning. *Exceptionality, 28*(5), 349-361.

Cawley, J. F., Kahn, H., & Tedesco, A. (1989). Vocational education and students with learning disabilities. *J Learn Disabil, 22*(10), 630-634, 640.

Chalita, P. J., Palacios, L., Cortes, J. F., Landeros-Weisenberger, A., Panza, K. E., & Bloch, M. H. (2012). Relationship of dropout and psychopathology in a high school sample in Mexico. *Front Psychiatry, 3*, 20.

Chin, J. L. (1976). Cognitive Tutoring as a Mental-Health Service for Children with Learning-Problems. *Professional Psychology, 7*(4), 518-524.

Cinar, H. U., Kizilkan, M. P., Akalin, A., Kiper, P. O. S., Utine, G. E., Derman, O., Kanbur, N., & Akgul, S. (2023). Assessing the Menstrual Cycle and Related Problems in Adolescents with a Genetic Syndrome Associated with Intellectual Disability. *Journal of Pediatric & Adolescent Gynecology, 06*, 06.

Classi, P., Milton, D., Ward, S., Sarsour, K., & Johnston, J. (2012). Social and emotional difficulties in children with ADHD and the impact on school attendance and healthcare utilization. *Child Adolesc Psychiatry Ment Health, 6*(1), 33.

Claussen, A. H., Bitsko, R. H., Holbrook, J. R., Bloomfield, J., & Giordano, K. (2018). Impact of Tourette Syndrome on School Measures in a Nationally Representative Sample. *J Dev Behav Pediatr, 39*(4), 335-342.

Clegg, J., Stackhouse, J., Finch, K., Murphy, C., & Nicholls, S. (2009). Language abilities of secondary age pupils at risk of school exclusion: A preliminary report. *Child Language Teaching & Therapy, 25*(1), 123-139.

Cooc, N. (2023). National Trends in Special Education and Academic Outcomes for English Learners With Disabilities. *Journal of Special Education*.

Corbett, W. P., Clark, H. B., & Blank, W. (2002). Employment and social outcomes associated with vocational programming for youths with emotional or behavioral disorders. *Behavioral Disorders, 27*(4), 358-370.

Cordoba, J., & Bagnato, M. J. (2021). Characterization of People with Functional Limitations from ICF Components Using the Longitudinal Social Protection Survey (ELPS) of Uruguay. *International Journal of Environmental Research and Public Health, 18*(15).

Correia-Zanini, M. R. G., Marturano, E. M., & Fontaine, A. M. G. V. (2018). Effects of early childhood education attendance on achievement, social skills, behaviour, and stress. *Estudos de Psicologia (Campinas), 35*(3), 287-297.

Cortez, E. G., & Malian, I. M. (2013). A Corrective Teaching Approach to Replace Undesired Behaviors in Students with Emotional and Behavioral Disorders. *Beyond Behavior, 22*(3), 54-59.

Coutinho, M. J., & Oswald, D. P. (1998). Understanding identification, placement and school completion rates for children with disabilities: The influence of economic, demographic and educational variables. *Advances in learning and behavioral disabilities, Vol. 12.*, 43-78.

Cumming, M. M., Criado, C., Park, J., Arango, A., Rodriguez, M. L., & Ali, M. (2023). Addressing Middle Schoolers' Disruptive Behavior: The Importance of Fostering Student Executive Functioning. *TEACHING Exceptional Children, 55*(3), 176-187.

Dabrowski, J., King, J., Edwards, K., Yates, R., Heyman, I., Zimmerman-Brenner, S., & Murphy, T. (2018). The Long-Term Effects of Group-Based Psychological Interventions for Children With Tourette Syndrome: A Randomized Controlled Trial. *Behav Ther, 49*(3), 331-343.

Dada, S., Andersson, A. K., May, A., Andersson, E. E., Granlund, M., & Huus, K. (2020). Agreement between participation ratings of children with intellectual disabilities and their primary caregivers. *Research in Developmental Disabilities, 104*.

Daniel, S. S., Walsh, A. K., Goldston, D. B., Arnold, E. M., Reboussin, B. A., & Wood, F. B. (2006). Suicidality, school dropout, and reading problems among adolescents. *J Learn Disabil, 39*(6), 507-514.

Daniels, B., Volpe, R. J., Fabiano, G. A., & Briesch, A. M. (2017). Classification accuracy and acceptability of the Integrated Screening and Intervention System Teacher Rating Form. *Sch Psychol Q, 32*(2), 212-225.

de la Barra, F. E., Vicente, B., Saldivia, S., & Melipillan, R. (2013). Epidemiology of ADHD in Chilean children and adolescents. *Atten Defic Hyperact Disord, 5*(1), 1-8.

deBettencourt, L. U., Zigmond, N., & Thornton, H. (1989). Follow-up of postsecondary-age rural learning disabled graduates and dropouts. *Except Child, 56*(1), 40-49.

Delk, J. L., Urbancik, G., Williams, C., Berg, G., & Kahn, M. W. (1974). Drop-outs from an American Indian reservation school: A possible prevention program. *Journal of Community Psychology, 2*(1), 15-17.

Dembo, R., Wareham, J., Krupa, J., & Winters, K. C. (2015). Sexual Risk Behavior among Male and Female Truant Youths: Exploratory, Multi-Group Latent Class Analysis. *J Alcohol Drug Depend, 3*(6), 1.

Dembo, R., Wareham, J., Schmeidler, J., & Winters, K. C. (2016). Exploratory two-level analysis of individual- and school-level factors on truant youth emotional/psychological functioning. *J Educ Res, 109*(6), 596-607.

Donahoe, K., & Zigmond, N. (1990). Academic Grades of Ninth-Grade Urban Learning-Disabled Students and Low-Achieving Peers. *Exceptionality: A Research Journal, 1*(1), 17-27.

Dopfner, M., Ise, E., Breuer, D., Rademacher, C., Metternich-Kaizman, T. W., & Schurmann, S. (2020). Long-Term Course After Adaptive Multimodal Treatment for Children With ADHD: An 8-Year Follow-Up. *J Atten Disord, 24*(1), 145-162.

Doren, B., Murray, C., & Gau, J. M. (2014). Salient Predictors of School Dropout among Secondary Students with Learning Disabilities. *Learning Disabilities Research & Practice, 29*(4), 150-159.

Dunn, C., Chambers, D., & Rabren, K. (2004). Variables affecting students' decisions to drop out of school. *Remedial and Special Education, 25*(5), 314-323.

Duquette, C., Stodel, E., Fullarton, S., & Hagglund, K. (2006). Teaching Students with Developmental Disabilities: Tips from Teens and Young Adults with Fetal Alcohol Spectrum Disorder. *TEACHING Exceptional Children, 39*(2), 28-31.

Edgar, E. (1987). Secondary programs in special education: Are many of them justifiable? *Special Issue: The transition from school to adult life, 53*(6), 555-561.

Edgington, R. E. (1975). SLD children: A ten-year follow-up. *Academic Therapy, 11*(1), 53-64.

Egalite, A. J. (2019). Peers with special educational needs and students' absences. *Educational Studies, 45*(2), 182-208.

Egger, H. L., Costello, E. J., & Angold, A. (2003). School refusal and psychiatric disorders: a community study. *J Am Acad Child Adolesc Psychiatry, 42*(7), 797-807.

Einat, T., & Einat, A. (2008). Learning disabilities and delinquency: a study of Israeli prison inmates. *Int J Offender Ther Comp Criminol, 52*(4), 416-434.

Einat, T., & Einat, A. (2015). To learn or not to learn-This is the question: Learning-disabled inmates' attitudes toward school, scholastic experiences, and the onset of criminal behavior. *The Prison Journal, 95*(4), 423-448.

Fergusson, D. M., & Horwood, L. J. (1995). Predictive validity of categorically and dimensionally scored measures of disruptive childhood behaviors. *J Am Acad Child Adolesc Psychiatry, 34*(4), 477-485; discussion 485-477.

Fergusson, D. M., Lynskey, M. T., & Horwood, L. J. (1995). Truancy in Adolescence. *New Zealand Journal of Educational Studies, 30*(1), 25-37.

Fichten, C. S., Nguyen, M. N., Amsel, R., Jorgensen, S., Budd, J., Jorgensen, M., Asuncion, J., & Barile, M. (2014). How Well Does the Theory of Planned Behavior Predict Graduation among College and University Students with Disabilities? *Social Psychology of Education: An International Journal, 17*(4), 657-685.

Filippello, P., Buzzai, C., Messina, G., & Mafodda, A. V., Sorrenti, L. (2019). School Refusal in Students with Low Academic Performances and Specific Learning Disorder. The Role of Self-Esteem and Perceived Parental Psychological Control. *International Journal of Disability Development and Education*.

Finning, K., Neochoriti Varvarrigou, I., Ford, T., Panagi, L., & Ukoumunne, O. C. (2022). Mental health and school absenteeism in children with long-term physical conditions: A secondary analysis of the British Child and Adolescent Mental Health Surveys 2004 and 2007. *Child: Care, Health & Development, 48*(1), 110-119.

Fleming, A. R., Plotner, A. J., & Oertle, K. M. (2017). College Students with Disabilities: The Relationship Between Student Characteristics, the Academic Environment, and Performance. *Journal of Postsecondary Education and Disability, 30*(3), 209-221.

Fleming, M., Fitton, C. A., Steiner, M. F. C., McLay, J. S., Clark, D., King, A., Mackay, D. F., & Pell, J. P. (2017). Educational and Health Outcomes of Children Treated for Attention-Deficit/Hyperactivity Disorder. *JAMA Pediatr, 171*(7), e170691.

Foster, M. E., Choo, A. L., & Smith, S. A. (2023). Speech-language disorder severity, academic success, and socioemotional functioning among multilingual and English children in the United States: The National Survey of Children's Health. *Frontiers in Psychology, 14*.

Franklin, C., & Streeter, C. L. (1992). Social support and psychoeducational interventions with middle class dropout youth. *Child & Adolescent Social Work Journal, 9*(2), 131-153.

Franklin, C., & Streeter, C. L. (1995). Assessment of Middle-Class Youth at-Risk to Dropout - School, Psychological and Family Correlates. *Children and Youth Services Review, 17*(3), 433-448.

Frater, J., Williams, I. G., & Hunter, C. (2018). Chromosome 7q11.23 duplication syndrome presenting as neuropsychiatric regression in late adolescence: A new manifestation of a new syndrome? *Aust N Z J Psychiatry, 52*(7), 715-716.

Fredriksen, M., Dahl, A. A., Martinsen, E. W., Klungsoyr, O., Faraone, S. V., & Peleikis, D. E. (2014). Childhood and persistent ADHD symptoms associated with educational failure and long-term occupational disability in adult ADHD. *Atten Defic Hyperact Disord, 6*(2), 87-99.

Fried, R., Petty, C., Faraone, S. V., Hyder, L. L., Day, H., & Biederman, J. (2016). Is ADHD a Risk Factor for High School Dropout? A Controlled Study. *J Atten Disord, 20*(5), 383-389.

Galloway, D. (1983). Research note: truants and other absentees. *J Child Psychol Psychiatry, 24*(4), 607-611.

Garcia Murillo, L., Ramos-Olazagasti, M. A., Mannuzza, S., Castellanos, F. X., & Klein, R. G. (2016). Childhood Attention-Deficit/Hyperactivity Disorder and Homelessness: A 33-Year Follow-Up Study. *J Am Acad Child Adolesc Psychiatry, 55*(11), 931-936.

Gillespie, J. (1982). The "pushouts": academic skills and learning disabilities in continuation high school students. *J Learn Disabil, 15*(9), 539-540.

Gonzalez, L. (2011). Class placement and academic and behavioral variables as predictors of graduation for students with disabilities. *Dissertation Abstracts International Section A: Humanities and Social Sciences, 71*(8-A), 2845.

Gore Langton, E., & Frederickson, N. (2016). Mapping the educational experiences of children with pathological demand avoidance. *Journal of Research in Special Educational Needs, 16*(4), 254-263.

Gottfried, M. A., Stiefel, L., Schwartz, A. E., & Hopkins, B. (2019). Showing Up: Disparities in Chronic Absenteeism Between Students With and Without Disabilities in Traditional Public Schools. *Teachers College Record, 121*(8).

Gregory, R. P., Hackney, C., & Gregory, N. M. (1982). Corrective Reading Program - an Evaluation. *British Journal of Educational Psychology, 52*(Feb), 33-50.

Gresham, F. M., Hunter, K. K., Corwin, E. P., & Fischer, A. J. (2013). Screening, Assessment, Treatment, and Outcome Evaluation of Behavioral Difficulties in an RTI Model. *Exceptionality, 21*(1), 19-33.

Grimes, S., Scevak, J., Southgate, E., & Buchanan, R. (2017). Non-disclosing students with disabilities or learning challenges: characteristics and size of a hidden population. *Australian Educational Researcher, 44*(4-5), 425-441.

Grimes, S., Southgate, E., Scevak, J., & Buchanan, R. (2021). Learning Impacts Reported by Students Living with Learning Challenges/Disability. *Studies in Higher Education, 46*(6), 1146-1158.

Hakkarainen, A. M., Holopainen, L. K., & Savolainen, H. K. (2015). A Five-Year Follow-Up on the Role of Educational Support in Preventing Dropout From Upper Secondary Education in Finland. *J Learn Disabil, 48*(4), 408-421.

Hancock, K. J., Cave, L., Christensen, D., Mitrou, F., & Zubrick, S. R. (2021). Associations Between Developmental Risk Profiles, Mental Disorders, and Student Absences Among Primary and Secondary Students in Australia. *School Mental Health, 13*(4), 756-771.

Hansen, C., Weiss, D., & Last, C. G. (1999). ADHD boys in young adulthood: psychosocial adjustment. *J Am Acad Child Adolesc Psychiatry, 38*(2), 165-171.

Harada, Y., Yamazaki, T., & Saitoh, K. (2002). Psychosocial problems in attention-deficit hyperactivity disorder with oppositional defiant disorder. *Psychiatry Clin Neurosci, 56*(4), 365-369.

Hassrick, E. M., Shih, W., Nuske, H. J., Vejnoska, S. F., Hochheimer, S., Linares, D. E., Ventimiglia, J., Carley, K., Stahmer, A. C., Smith, T., Mandell, D., & Kasari, C. (2021). Disrupted Care Continuity: Testing Associations between Social Networks and Transition Success for Children with Autism. *Social Sciences, 10*(7).

Hayden, L. K., & McLaughlin, T. F. (1987). Effects of a study skills curriculum with rural high school learning disabled students. *Techniques, 3*(3), 162-171.

Henson, K. S. (2018). Students with disabilities at risk: Predictors of on-time graduation. *Dissertation Abstracts International Section A: Humanities and Social Sciences, 79*(1-A(E)), No-Specified.

Herron, M. D., & Martin, J. (2015). Capacity and Opportunity: Predicting Engagement for Middle School Students With Behavioral Disorders. *Journal of Emotional and Behavioral Disorders, 23*(4), 215-225.

Higgins, C. (2019). Southwestern Illinois teachers' perceptions of students with learning disabilities and/or ADHD transitioning to high school. *Dissertation Abstracts International Section A: Humanities and Social Sciences, 80*(8-A(E)), No-Specified.

Hinojosa, M. S., Fernandez-Baca, D., & Knapp, C. (2012). Factors associated with family-provider partnership among children with ADHD. *Fam Med, 44*(7), 463-470.

Hogan, A., McLellan, L., & Bauman, A. (2000). Health promotion needs of young people with disabilities--a population study. *Disabil Rehabil, 22*(8), 352-357.

Hollar, D., & Moore, D. (2004). Relationship of substance use by students with disabilities to long-term educational, employment, and social outcomes. *Subst Use Misuse, 39*(6), 931-962.

Hoyle, J. N., Laditka, J. N., & Laditka, S. B. (2020). Severe developmental disability and the transition to adulthood. *Disability & Health Journal, 13*(3), 100912.

Hughes, A., Wade, K. H., Dickson, M., Rice, F., Davies, A., Davies, N. M., & Howe, L. D. (2021). Common health conditions in childhood and adolescence, school absence, and educational attainment: Mendelian randomization study. *NPJ Science of Learning, 6*(1), 1.

Hysing, M., Petrie, K. J., Boe, T., & Sivertsen, B. (2017). Parental work absenteeism is associated with increased symptom complaints and school absence in adolescent children. *BMC Public Health, 17*(1), 439.

Ishii, T., Takahashi, O., Kawamura, Y., & Ohta, T. (2003). Comorbidity in attention deficit-hyperactivity disorder. *Psychiatry Clin Neurosci, 57*(5), 457-463.

Islam, M. S., Rashid, M. H., Uddin, M. N., Singha, R. K., Rahman, M. A., Haque, M. A., Saha, C. K., & Abedin, M. F. (2017). Psychiatric Disorders in Drop out from Educational Attainment Attending Mental Health Facilities: A Descriptive Cross Sectional Study. *Mymensingh Med J, 26*(3), 551-557.

Jackson, S. L. J., Hart, L., Brown, J. T., & Volkmar, F. R. (2018). Brief Report: Self-Reported Academic, Social, and Mental Health Experiences of Post-Secondary Students with Autism Spectrum Disorder. *J Autism Dev Disord, 48*(3), 643-650.

Jansen, D., Petry, K., Ceulemans, E., Noens, I., & Baeyens, D. (2016). Functioning and participation problems of students with ASD in higher education: which reasonable accommodations are effective? *European Journal of Special Needs Education, 32*(1), 71-88.

January, S. A. A., Lambert, M. C., Epstein, M. H., Spooner, M., & Gebreselassie, T. (2018). Students at Enrollment Into Community-Based Systems of Care: Characteristics and Predictors of Functioning in School. *Journal of Emotional and Behavioral Disorders, 26*(2), 67-78.

Johnsen, D. B., Lomholt, J. J., Heyne, D., Jeppesen, P., Jensen, M. B., Silverman, W. K., & Thastum, M. (2022). Sociodemographic and clinical characteristics of youths and parents seeking psychological treatment for school attendance problems. *PLoS ONE, 17*(1).

Kano, Y., Ohta, M., Nagai, Y., & Scahill, L. (2010). Association between Tourette syndrome and comorbidities in Japan. *Brain Dev, 32*(3), 201-207.

Karande, S., Gogtay, N. J., Shaikh, N., Sholapurwala, R., More, T., & Meshram, P. (2023). Self-perceived anxiety symptoms in school students with borderline intellectual functioning: A cross-sectional questionnaire-based study in Mumbai, Maharashtra, India. *Journal of Postgraduate Medicine, 69*(2), 89-96.

Karpinski, M. J., Neubert, D. A., & Graham, S. (1992). A follow-along study of postsecondary outcomes for graduates and dropouts with mild disabilities in a rural setting. *J Learn Disabil, 25*(6), 376-385.

Kearney, C. A., & Albano, A. M. (2004). The functional profiles of school refusal behavior. Diagnostic aspects. *Behav Modif, 28*(1), 147-161.

Kellogg, E., & McLaughlin, F. T. (1997). A Comparison of Mid-Year Holding Power between Tenth Grade General and Special Education Programs. *B.C. Journal of Special Education, 21*(1), 31-46.

Kent, K. M., Pelham, W. E., Jr., Molina, B. S., Sibley, M. H., Waschbusch, D. A., Yu, J., Gnagy, E. M., Biswas, A., Babinski, D. E., & Karch, K. M. (2011). The academic experience of male high school students with ADHD. *J Abnorm Child Psychol, 39*(3), 451-462.

Knapp, C. A., Hinojosa, M., Baron-Lee, J., Fernandez-Baca, D., Hinojosa, R., & Thompson, L. (2012). Factors associated with a medical home among children with Attention-Deficit Hyperactivity Disorder. *Matern Child Health J, 16*(9), 1771-1778.

Kishore, A. N., & Shaji, K. S. (2012). School dropouts: examining the space of reasons. *Indian J Psychol Med, 34*(4), 318-323.

Kissman, K. (1998). High risk behavior among adolescent mothers: The problem in context. *Early Child Development and Care, 143*, 103-112.

Kolvin, I., Garside, R. F., Nicol, A. R., Leitch, I., & Macmillan, A. (1977). Screening schoolchildren for high risk of emotional and educational disorder. *Br J Psychiatry, 131*, 192-206.

Korematsu, S., Takano, T., & Izumi, T. (2016). Pre-school development and behavior screening with a consecutive support programs for 5-year-olds reduces the rate of school refusal. *Brain Dev, 38*(4), 373-376.

Korhonen, J., Linnanmaki, K., & Aunio, P. (2014). Learning difficulties, academic well-being and educational dropout: A person-centred approach. *Learning and Individual Differences, 31*, 1-10.

Kortering, L., Haring, N., & Klockars, A. (1992). The identification of high-school dropouts identified as learning disabled: evaluating the utility of a discriminant analysis function. *Except Child, 58*(5), 422-435.

Kortering, L. J., & Braziel, P. M. (1998). School Dropout among Youth with and without Learning Disabilities. *Career Development for Exceptional Individuals, 21*(1), 61-74.

Kortering, L. J., & Braziel, P. M. (1999). Staying in school - The perspective of ninth-grade students. *Remedial and Special Education, 20*(2), 106-113.

Kortering, L. J., Braziel, P. M., & Tompkins, J. R. (2002). The challenge of school completion among youths with behavioral disorders: Another side of the story. *Behavioral Disorders, 27*(2), 142-154.

Kortering, L. L., & Blackorby, J. (1992). High school dropout and students identified with behavioral disorders. *Behavioral Disorders, 18*(1), 24-32.

Kotkov, B. (1966). Disposition of the school leaver. *Dis Nerv Syst, 27*(3), 178-182.

Kunzweiler, C., Franciosa, J., & Brown, H. (1976). Adult education as an alternative for the emotionally disturbed. *Journal of Instructional Psychology, 3*(4), 2-4.

Kunzweiler, C., Franciosa, J., & Brown, H. (1977). Adult education as an alternative for the emotionally disturbed. *Education, 98*(1), 72-74.

Kurita, H. (1988). A case of Heller's syndrome with school refusal. *J Autism Dev Disord, 18*(2), 315-319.

Langenkamp, A. G. (2016). Effects of School Mobility on Adolescent Social Ties and Academic Adjustment. *Youth & Society, 48*(6), 810-833.

Lawrence, D., Dawson, V., Houghton, S., Goodsell, B., & Sawyer, M. G. (2019). Impact of mental disorders on attendance at school. *Australian Journal of Education, 63*(1), 5-21.

Lawrence, D., Hafekost, J., Johnson, S. E., Saw, S., Buckingham, W. J., Sawyer, M. G., Ainley, J., & Zubrick, S. R. (2016). Key findings from the second Australian Child and Adolescent Survey of Mental Health and Wellbeing. *Aust N Z J Psychiatry, 50*(9), 876-886.

Lax, Y., Brown, S. N., Silver, M., & Brown, N. M. (2021). Associations Between Participation in After-School Activities, Attention-Deficit/Hyperactivity Disorder Severity, and School Functioning. *Journal of Developmental & Behavioral Pediatrics, 42*(4), 257-263.

Lazerson, D. B., Foster, H. L., Brown, S. I., & Hummel, J. W. (1988). The effectiveness of cross-age tutoring with truant, junior high school students with learning disabilities. *J Learn Disabil, 21*(4), 253-255.

Lemaire, G. S., Mallik, K., & Stoll, B. G. (2002). Expanding horizons: A model academic and vocational training program for out-of-school youth with disabilities. *Journal of Rehabilitation, 68*(2), 39-45.

Leone, P., Lovitt, T. C., & Hansen, C. R. (1981). A descriptive followup study of learning disabled boys. *Learning Disability Quarterly, 4*(2), 152-162.

Lessard, A., Butler-Kisber, L., Fortin, L., & Marcotte, D. (2014). Analyzing the Discourse of Dropouts and Resilient Students. *Journal of Educational Research, 107*(2), 103-110.

Levin, E. K., Zigmond, N., & Birch, J. W. (1985). A follow-up study of 52 learning disabled adolescents. *J Learn Disabil, 18*(1), 2-7.

Levy, S. R., Perhats, C., Nash-Johnson, M., & Welter, J. F. (1992). Reducing the risks in pregnant teens who are very young and those with mild mental retardation. *Ment Retard, 30*(4), 195-203.

Lichtenstein, S. (1993). Transition from school to adulthood: case studies of adults with learning disabilities who dropped out of school. *Except Child, 59*(4), 336-347.

Lindly, O. J., Martin, A. J., & Lally, K. (2020). A Profile of Care Coordination, Missed School Days, and Unmet Needs Among Oregon Children with Special Health Care Needs with Behavioral and Mental Health Conditions. *Community Mental Health Journal, 56*(8), 1571-1580.

Linet, L. S. (1985). Tourette syndrome, pimozide, and school phobia: the neuroleptic separation anxiety syndrome. *Am J Psychiatry, 142*(5), 613-615.

Locquiao, J., Abernathy, T. V., Mesina, O. L., & Liu, L. P. (2021) The Relationship Between Absences and Passing Tests for Students With and Without Specific Learning Disabilities. *Journal of Education for Students Placed at Risk, 26*(3), 253-278.

Maher, C. A. (1982). Behavioral-Effects of Using Conduct Problem Adolescents as Cross-Age Tutors. *Psychology in the Schools, 19*(3), 360-364.

Mandel, D. S., Walrath, C. M., Manteuffel, B., Sgro, G., & Pinto-Martin, J. (2005). Characteristics of Children with Autistic Spectrum Disorders Served in Comprehensive Community-based Mental Health Settings. *Journal of Autism and Developmental Disorders, 35*(3), 313-321.

Martin, A. J. (2014). The role of ADHD in academic adversity: disentangling ADHD effects from other personal and contextual factors. *Sch Psychol Q, 29*(4), 395-408.

Mallett, C. A., Quinn, L., Yun, J., & Fukushima-Tedor, M. (2022). The "Learning Disabilities-to-Prison" Pipeline: Evidence From the Add Health National Longitudinal Study. *Crime & Delinquency*.

Maughan, B., Gray, G., & Rutter, M. (1985). Reading retardation and antisocial behaviour: a follow-up into employment. *J Child Psychol Psychiatry, 26*(5), 741-758.

Maughan, B., Hagell, A., Rutter, M., & Yule, W. (1994). Poor Readers in Secondary-School. *Reading and Writing, 6*(2), 125-150.

Maughan, B., Pickles, A., Hagell, A., Rutter, M., & Yule, W. (1996). Reading problems and antisocial behaviour: developmental trends in comorbidity. *J Child Psychol Psychiatry, 37*(4), 405-418.

May, F., Ford, T., Janssens, A., Newlove-Delgado, T., Emma Russell, A., Salim, J., Ukoumunne, O. C., & Hayes, R. (2021). Attainment, attendance, and school difficulties in UK primary schoolchildren with probable ADHD. *British Journal of Educational Psychology, 91*(1), 442-462.

McConnell, F., Horton, K. B., & Smith, B. R. (1969). Language development and cultural disadvantagement. *Exceptional Children, 35*(8), 597-606.

Mekonnen, H., Medhin, G., Tomlinson, M., Alem, A., Prince, M., & Hanlon, C. (2020). Impact of child emotional and behavioural difficulties on educational outcomes of primary school children in Ethiopia: A population-based cohort study. *Child and Adolescent Psychiatry and Mental Health, 14*.

Merga, M. K. (2019). “Fallen through the cracks”: Teachers’ perceptions of barriers faced by struggling literacy learners in secondary school. *English in Education*, 1-25.

Meyer, E. A., Hagopian, L. P., & Paclawskyj, T. R. (1999). A function-based treatment for school refusal behavior using shaping and fading. *Res Dev Disabil, 20*(6), 401-410.

Mikkelsen, E. J., Detlor, J., & Cohen, D. J. (1981). School avoidance and social phobia triggered by haloperidol in patients with Tourette's disorder. *Am J Psychiatry, 138*(12), 1572-1576.

Mills, B., & Sabornie, E. Variables that Predict Graduation for Students with Emotional Disturbance: An examination Across High Schools. *International Journal of Disability Development and Education*.

Milsom, A., & Glanville, J. L. (2010). Factors Mediating the Relationship Between Social Skills and Academic Grades in a Sample of Students Diagnosed With Learning Disabilities or Emotional Disturbance. *Remedial and Special Education, 31*(4), 241-251.

Minde, K., Lewin, D., Weiss, G., Lavigueur, H., Douglas, V., & Sykes, E. (1971). The hyperactive child in elementary school: A 5 year, controlled, followup. *Exceptional Children, 38*(3), 215-221.

Moccia, R. E., & et al. (1989). A Mentor Program for Facilitating the Life Transitions of Individuals Who Have Handicapping Conditions. *Journal of Reading, Writing, and Learning Disabilities International, 5*(2), 177-195.

Montague, M., Enders, C., & Castro, M. (2005). Academic and behavioral outcomes for students at risk for emotional and behavioral disorders. *Behavioral Disorders, 31*(1), 84-94.

Muhammad, N. A., Wan Ismail, W. S., Tan, C. E., Jaffar, A., Sharip, S., & Omar, K. (2011). Attention-deficit hyperactive disorder presenting with school truancy in an adolescent: a case report. *Ment Health Fam Med, 8*(4), 249-254.

Mullen, F. A. (1950). Truancy and classroom disorder as symptoms of personality problems. *Journal of Educational Psychology, 41*(2), 97-109.

Murphy, K., & Barkley, R. A. (1996). Attention deficit hyperactivity disorder adults: comorbidities and adaptive impairments. *Compr Psychiatry, 37*(6), 393-401.

Naylor, M. W., Staskowski, M., Kenney, M. C., & King, C. A. (1994). Language disorders and learning disabilities in school-refusing adolescents. *J Am Acad Child Adolesc Psychiatry, 33*(9), 1331-1337.

Niemi, S., Lagerstrom, M., & Alanko, K. (2022). School attendance problems in adolescent with attention deficit hyperactivity disorder. *Frontiers in Psychology, 13*, 1017619.

Nissinen, N. M., Gissler, M., Sarkola, T., Kahila, H., Autti-Ramo, I., & Koponen, A. M. (2021). Completed secondary education among youth with prenatal substance exposure: A longitudinal register-based matched cohort study. *Journal of Adolescence, 86*, 15-27.

Onofri, A., Olivieri, L., Silva, P., Bernassola, M., & Tozzi, E. (2021). Correlation between primary headaches and learning disabilities in children and adolescents. *Minerva Pediatrics, 15*, 15.

Orm, S., Orm, C., Mebostad, M. I., Dechsling, A., & Nordahl-Hansen, A. (2022). Confirming the Validity of the School-Refusal Assessment Scale-Revised in a Sample of Children With Attention-Deficit/Hyperactivity Disorder. *Frontiers in Psychology, 13*, 849303.

Pagani, L. S., & Fitzpatrick, C. (2016). Early Childhood Household Smoke Exposure Predicts Less Task-Oriented Classroom Behavior at Age 10. *Health Educ Behav, 43*(5), 584-591.

Palfiova, M., Dankulincova Veselska, Z., Bobakova, D., Holubcikova, J., Cermak, I., Madarasova Geckova, A., van Dijk, J. P., & Reijneveld, S. A. (2017). Is risk-taking behaviour more prevalent among adolescents with learning disabilities? *Eur J Public Health, 27*(3), 501-506.

Peguero, A. A., Merrin, G. J., Hong, J. S., & Johnson, K. R. (2019). School Disorder and Dropping Out: The Intersection of Gender, Race, and Ethnicity. *Youth & Society, 51*(2), 193-218.

Pickard, K., Meyer, A., Reyes, N., Tanda, T., & Reaven, J. (2021). Using evaluative frameworks to examine the implementation outcomes of a cognitive behavioral therapy program for autistic students with anxiety within public school settings. *Autism*, 13623613211065797.

Pingault, J. B., Tremblay, R. E., Vitaro, F., Carbonneau, R., Genolini, C., Falissard, B., & Cote, S. M. (2011). Childhood trajectories of inattention and hyperactivity and prediction of educational attainment in early adulthood: a 16-year longitudinal population-based study. *Am J Psychiatry, 168*(11), 1164-1170.

Plapp, J. M. (1990). Tourettes and school refusal. *J Am Acad Child Adolesc Psychiatry, 29*(1), 149-150.

Plasman, J. S. (2018). Career/Education Plans and Student Engagement in Secondary School. *American Journal of Education, 124*(2), 217-246.

Plasman, J. S., & Gottfried, M. A. (2018). Applied STEM Coursework, High School Dropout Rates, and Students With Learning Disabilities. *Educational Policy, 32*(5), 664-696.

Polirstok, S. R. (1987). Ecological effects of home-based, school-based, and community-based training of parents of children with learning and behavior problems. *Int J Rehabil Res, 10*(3), 293-301.

Potthoff, J.-O. (1979). Late Again? Three Techniques to Reduce Tardiness in Secondary Learning Handicapped Students. *TEACHING Exceptional Children, 11*(4), 146-148.

Pratinidhi, A. K., Kurulkar, P. V., Garad, S. G., & Dalal, M. (1992). Epidemiological aspects of school dropouts in children between 7-15 years in rural Maharashtra. *Indian J Pediatr, 59*(4), 423-427.

Ramirez, C. A., Rosen, L. A., Deffenbacher, J. L., Hurst, H., Nicoletta, C., Rosencranz, T., & Smith, K. (1997). Anger and anger expression in adults with high ADHD symptoms. *Journal of Attention Disorders, 2*(2), 115-128.

Ramos-Galarza, C., Fiallo-Karolys, X., Ramos, V., Jadán-Guerrero, J., & Paredes-Núñez, L. (2018). Attention deficit hyperactivity disorder: Behavioral report from professors and self-report from university students. *Psychology & Neuroscience, 11*(1), 95-104.

Rayle, J. M. (1998). The exploration alternative school: A case study. *The High School Journal, 81*(4), 244-250.

Rea, P. J., McLaughlin, V. L., & Walther-Thomas, C. (2002). Outcomes for students with learning disabilities in inclusive and pullout programs. *Exceptional Children, 68*(2), 203-222.

Redmond, S. M., & Hosp, J. L. (2008). Absenteeism rates in students receiving services for CDs, LDs, and EDs: a macroscopic view of the consequences of disability. *Lang Speech Hear Serv Sch, 39*(1), 97-103.

Reed, M. O., Jakubovski, E., Johnson, J. A., & Bloch, M. H. (2017). Predictors of Long-Term School-Based Behavioral Outcomes in the Multimodal Treatment Study of Children with Attention-Deficit/Hyperactivity Disorder. *J Child Adolesc Psychopharmacol, 27*(4), 296-309.

Reissner, V., Jost, D., Krahn, U., Knollmann, M., Weschenfelder, A. K., Neumann, A., Wasem, J., & Hebebrand, J. (2015). The Treatment of School Avoidance in Children and Adolescents With Psychiatric Illness. *Dtsch Arztebl Int, 112*(39), 655-662.

Reschly, A. L., & Christenson, S. L. (2006). Prediction of dropout among students with mild disabilities - A case for the inclusion of student engagement variables. *Remedial and Special Education, 27*(5), 276-292.

Ripley, K., & Yuill, N. (2005). Patterns of language impairment and behaviour in boys excluded from school. *Br J Educ Psychol, 75*(Pt 1), 37-50.

Roetman, P. J., Lundstrom, S., Finkenauer, C., Vermeiren, R., Lichtenstein, P., & Colins, O. F. (2019). Children With Early-Onset Disruptive Behavior: Parental Mental Disorders Predict Poor Psychosocial Functioning in Adolescence. *J Am Acad Child Adolesc Psychiatry, 58*(8), 806-817.

Rosas, R., Escobar, J.-P., Ramírez, M.-P., Meneses, A., & Guajardo, A. (2017). Impact of a computer-based intervention in Chilean children at risk of manifesting reading difficulties / Impacto de una intervención basada en ordenador en niños chilenos con riesgo de manifestar dificultades lectoras. *Infancia y Aprendizaje, 40*(1), 158-188.

Roue, A., Harf, A., Benoit, L., Sibeoni, J., & Moro, M. R. (2021). Multifamily Therapy for Adolescents With School Refusal: Perspectives of the Adolescents and Their Parents. *Frontiers in psychiatry Frontiers Research Foundation, 12*, 624841.

Rubin, D. (1974). Inner-city high school teachers as teachers of reading: A possible solution to the dropout problem. *Journal of Negro Education, 43*(3), 337-347.

Russell, T., & Boswell, C. F. (1986). A comparison of selected demographic, educational, and behavioral factors among adolescent mentally retarded offenders and adolescent mentally retarded nonoffenders. *Journal of Offender Counseling, Services & Rehabilitation, 10*(3), 5-24.

Safer, D. J., Heaton, R. C., & Parker, F. C. (1981). A behavioral program for disruptive Junior High school students: results and follow-up. *J Abnorm Child Psychol, 9*(4), 483-494.

Saraiva, A. B., Pereira, B. O., & Zamith-Cruz, J. (2011). School dropout, problem behaviour and poor academic achievement: A longitudinal view of Portuguese male offenders. *Emotional & Behavioural Difficulties, 16*(4), 419-436.

Sasser, T. R., Kalvin, C. B., & Bierman, K. L. (2016). Developmental trajectories of clinically significant attention-deficit/hyperactivity disorder (ADHD) symptoms from grade 3 through 12 in a high-risk sample: Predictors and outcomes. *J Abnorm Psychol, 125*(2), 207-219.

Satila, H., Jolma, L. M., Merilainen-Nipuli, M., & Koivu-Jolma, M. (2022). Challenges and Neuropsychological Functioning in Children and Adolescents with Borderline Intellectual Functioning. *Children-Basel, 9*(12).

Scanlon, D., & Mellard, D. F. (2002). Academic and participation profiles of school-age dropouts with and without disabilites. *Exceptional Children, 68*(2), 239-258.

Schloss, P. J., Kane, M. S., & Miller, S. R. (1981). Truancy intervention with behavior disordered adolescents. *Behavioral Disorders, 6*(3), 175-179.

Schmengler, H., Peeters, M., Stevens, G., Hartman, C. A., Oldehinkel, A. J., & Vollebergh, W. A. M. (2023). ADHD Symptoms and Educational Level in Adolescents: The Role of the Family, Teachers, and Peers. *Research on Child and Adolescent Psychopathology, 23*, 23.

Schmidt, J. J., & Biles, J. W. (1985). Puppetry as a group counseling technique with middle school students. *Elementary School Guidance & Counseling, 20*(1), 67-73.

Schmidt, L. B., Corn, G., Wohlfahrt, J., Melbye, M., & Munch, T. N. (2018). School performance in children with infantile hydrocephalus: a nationwide cohort study. *Clin Epidemiol, 10*, 1721-1731.

Schwartz, A. E., Hopkins, B. G., & Stiefel, L. The Effects of Special Education on the Academic Performance of Students with Learning Disabilities. *Journal of Policy Analysis and Management*.

Seidel, J. F., & Vaughn, S. (1991). Social alienation and the learning disabled school dropout. *Learning Disabilities Research & Practice, 6*(3), 152-157.

Sentenac, M., Lach, L. M., Gariepy, G., & Elgar, F. J. (2019). Education disparities in young people with and without neurodisabilities. *Dev Med Child Neurol, 61*(2), 226-231.

Shechtman, Z. (1993). School Adjustment and Small-Group Therapy - an Israeli Study. *Journal of Counseling and Development, 72*(1), 77-81.

Shier, V., Nicosia, N., Shih, R., & Datar, A. (2019). Ambient air pollution and children's cognitive outcomes. *Population and Environment, 40*(3), 347-367.

Sibley, M. H., Morley, C., Rodriguez, L., Coxe, S. J., Evans, S. W., Morsink, S., & Torres, F. (2020). A peer-delivered intervention for high school students with impairing ADHD symptoms. *School Psychology Review, 49*(3), 275-290.

Sigman, M., Neumann, C., Jansen, A. A., & Bwibo, N. (1989). Cognitive abilities of Kenyan children in relation to nutrition, family characteristics, and education. *Child Development, 60*(6), 1463-1474.

Sinclair, I., Luke, N., & Berridge, D. (2019). Children in Care or in Need: Educational Progress at Home and in Care. *Oxford Review of Education, 45*(4), 443-460.

Sinclair, M. F., Christenson, S. L., Evelo, D. L., & Hurley, C. M. (1998). Dropout prevention for youth with disabilities: Efficacy of a sustained school engagement procedure. *Exceptional Children, 65*(1), 7-21.

Sinclair, M. F., Christenson, S. L., & Thurlow, M. L. (2005). Promoting school completion of urban secondary youth with emotional or behavioral disabilities. *Exceptional Children, 71*(4), 465-482.

Sitlington, P. L., & Frank, A. R. (1993). Dropouts with learning disabilities: What happens to them as young adults? *Learning Disabilities Research & Practice, 8*(4), 244-252.

Skedgell, K., & Kearney, C. A. (2016). Predictors of Absenteeism Severity in Truant Youth: A Dimensional and Categorical Analysis. *American Secondary Education, 45*(1), 46-58.

Smart, D., Youssef, G. J., Sanson, A., Prior, M., Toumbourou, J. W., & Olsson, C. A. (2019). Social mediators of relationships between childhood reading difficulties, behaviour problems, and secondary school noncompletion. *Australian Journal of Psychology, 71*(2), 171-182.

Smith-Fromm, T., & Evans-Agnew, R. A. (2017). Educator Preparedness for Mental Health in Adolescents: Opportunities for School Nurse Leadership. *NASN Sch Nurse, 32*(6), 372-377.

Smith, I., Beasley, M. G., Wolff, O. H., & Ades, A. E. (1988). Behavior disturbance in 8-year-old children with early treated phenylketonuria. Report from the MRC/DHSS Phenylketonuria Register. *J Pediatr, 112*(3), 403-408.

Smith, T. S., Manuel, N., & Stokes, B. R. (2012). Comparisons of High School Graduation Rates of Students with Disabilities and Their Peers in Twelve Southern States. *Learning Disabilities: A Multidisciplinary Journal, 18*(2), 47-59.

Snowling, M. J., Adams, J. W., Bowyer-Crane, C., & Tobin, V. (2000). Levels of literacy among juvenile offenders: The incidence of specific reading difficulties. *Criminal Behaviour and Mental Health, 10*(4), 229-241.

Snyder, J. J., & White, M. J. (1979). Use of Cognitive Self-Instruction in the Treatment of Behaviorally Disturbed Adolescents. *Behavior Therapy, 10*(2), 227-235.

Soendergaard, H. M., Thomsen, P. H., Pedersen, P., Pedersen, E., Poulsen, A. E., Nielsen, J. M., Winther, L., Henriksen, A., Rungoe, B., & Soegaard, H. J. (2016). Treatment dropout and missed appointments among adults with attention-deficit/hyperactivity disorder: associations with patient- and disorder-related factors. *J Clin Psychiatry, 77*(2), 232-239.

Solmaz, M., Belli, H., & Saygili, S. (2011). An adolescent case with internet addiction and hacking: How are we dealing with this diverse spectrum of disorder? *General Hospital Psychiatry, 33*(4), e15-e16.

Soul, O., Gross, R., Basel, D., Mosheva, M., Kushnir, J., Efron, M., Dekel, I., Weizman, A., & Gothelf, D. (2021). Stimulant Treatment Effect on Anxiety Domains in Children with Attention-Deficit/Hyperactivity Disorder With and Without Anxiety Disorders: A 12-Week Open-Label Prospective Study. *Journal of Child & Adolescent Psychopharmacology, 31*(9), 639-644.

Souto, B. K. (1996). A parent's perspective on transition to adult life for youth, identifed as seriously emotionally disturbed. *Community Alternatives: International Journal of Family Care, 8*(2), 26-32.

Stattin, H., & Magnusson, D. (1995). Onset of official delinquency: Its co-occurence in time with educational, behavioural, and interpersonal problems. *British Journal of Criminology, 35*(3), 417-449.

Stein, K. F., Connors, E. H., Chambers, K. L., Thomas, C. L., & Stephan, S. H. (2016). Youth, Caregiver, and Staff Perspectives on an Initiative to Promote Success of Emerging Adults with Emotional and Behavioral Disabilities. *J Behav Health Serv Res, 43*(4), 582-596.

Stewart, S. L., Klassen, J., & Hamza, C. (2016). Emerging mental health diagnoses and school disruption: An examination among clinically referred children and youth. *Exceptionality Education International, 26*(2), 5-20.

Strand, A.-S. M., & Granlund, M. (2014). The school situation for students with a high level of absenteeism in compulsory school: Is there a pattern in documented support? *Scandinavian Journal of Educational Research, 58*(5), 551-569.

Sullivan, P. M., & Knutson, J. F. (2000). The prevalence of disabilities and maltreatment among runaway children. *Child Abuse Negl, 24*(10), 1275-1288.

Sung, V., Hiscock, H., Sciberras, E., & Efron, D. (2008). Sleep problems in children with attention-deficit/hyperactivity disorder: prevalence and the effect on the child and family. *Arch Pediatr Adolesc Med, 162*(4), 336-342.

Svensson, I., Lundberg, I., & Jacobson, C. (2003). The nature of reading difficulties among inmates in juvenile institutions. *Reading and Writing, 16*(7), 667-691.

Talbott, E., Fleming, J., Karabatsos, G., & Dobria, L. (2011). Making Sense of Minority Student Identification in Special Education: School Context Matters. *International Journal of Special Education, 26*(3), 150-170.

Tansey, T. N., Smedema, S., Umucu, E., Iwanaga, K., Wu, J. R., Cardoso, E. D., & Strauser, D. (2018). Assessing College Life Adjustment of Students With Disabilities: Application of the PERMA Framework. *Rehabilitation Counseling Bulletin, 61*(3), 131-142.

Theobald, R. J., Goldhaber, D. D., Gratz, T. M., & Holden, K. L. (2018). Career and Technical Education, Inclusion, and Postsecondary Outcomes for Students With Learning Disabilities. *J Learn Disabil*, 22219418775121.

Toomey, S. L., Chan, E., Ratner, J. A., & Schuster, M. A. (2011). The patient-centered medical home, practice patterns, and functional outcomes for children with attention deficit/hyperactivity disorder. *Acad Pediatr, 11*(6), 500-507.

Tramontina, S., Martins, S., Michalowski, M. B., Ketzer, C. R., Eizirik, M., Biederman, J., & Rohde, L. A. (2002). Estimated mental retardation and school dropout in a sample of students from state public schools in Porto Alegre, Brazil. *Revista Brasileira de Psiquiatria, 24*(4), 177-181.

Trampush, J. W., Miller, C. J., Newcorn, J. H., & Halperin, J. M. (2009). The impact of childhood ADHD on dropping out of high school in urban adolescents/ young adults. *J Atten Disord, 13*(2), 127-136.

Troiano, P. F., Liefeld, J. A., & Trachtenberg, J. V. (2010). Academic Support and College Success for Postsecondary Students with Learning Disabilities. *Journal of College Reading and Learning, 40*(2), 35-44.

Uiters, E., Maurits, E., Droomers, M., Zwaanswijk, M., Verheij, R. A., & van der Lucht, F. (2014). The association between adolescents' health and disparities in school career: a longitudinal cohort study. *BMC Public Health, 14*, 1104.

Vardanian, M. M., Scavenius, C., Granski, M., & Chacko, A. (2019). An International Examination of the Effectiveness of Functional Family Therapy (FFT) in a Danish Community Sample. *J Marital Fam Ther, 13*, 13.

Vardanian, M. M., Scavenius, C., Granski, M., & Chacko, A. (2020). An International Examination of the Effectiveness of Functional Family Therapy (FFT) in a Danish Community Sample. *Journal of Marital & Family Therapy, 46*(2), 289-303.

Vassilopoulos, A., N, L. P., & Ibeziako, P. (2021). School absenteeism as a potential proxy of functionality in pediatric patients with somatic symptom and related disorders. *Clinical Child Psychology & Psychiatry, 26*(2), 342-354.

Vish, N. L., & Stolfi, A. (2020). Relationship of Children's Emotional and Behavioral Disorders With Health Care Utilization and Missed School. *Academic pediatrics, 20*(5), 687-695.

Vnukova, M., Dechterenko, F., Weissenberger, S., Anders, M., & Ptacek, R. (2023). Childhood School Performance in Adults Diagnosed with Attention-Deficit/Hyperactivity Disorder. *Journal of Attention Disorders, 27*(3), 307-312.

Vogel, S. A., & Holt, J. K. (2003). A comparative study of adults with and without self-reported learning disabilities in six English-speaking populations: what have we learned? *Dyslexia, 9*(4), 193-228.

Vukic, V. V., & Zrilic, S. (2016). The Connection between Pupils' School Success and Their Inclusiveness in Extracurricular and Out-of-School Activities in Croatia. *World Journal of Education, 6*(3), 29-38.

Wagner, M. M., & Blackorby, J. (1996). Transition from high school to work or college: how special education students fare. *Future Child, 6*(1), 103-120.

Waldron, K. A. (1985). The effects of an intermediary placement on learning disabled and low-achieving adolescents. *J Learn Disabil, 18*(3), 154-159.

Wall-Wieler, E., Roos, L. L., Chateau, D. G., & Rosella, L. C. (2016). What predictors matter: Risk factors for late adolescent outcomes. *Can J Public Health, 107*(1), e16-e22.

Webster-Stratton, C., & Reid, M. J. (2003). Treating conduct problems and strengthening social and emotional competence in young children: The Dina Dinosaur treatment program. *Journal of Emotional and Behavioral Disorders, 11*(3), 130-143.

Weimer, D. L., Moberg, P., French, F., Tanner-Smith, E. E., & Finch, A. J. (2019). Net Benefits of Recovery High Schools: Higher Cost but Increased Sobriety and Educational Attainment. *J Ment Health Policy Econ, 22*(3), 109-120.

Weitzman, M., Walker, D. K., & Gortmaker, S. (1986). Chronic illness, psychosocial problems, and school absences. Results of a survey of one county. *Clin Pediatr (Phila), 25*(3), 137-141.

Whitley, J., Lupart, J., & Beran, T. (2007). The characteristics and experiences of Canadian students receiving special education services for a learning disability. *Exceptionality Education Canada, 17*(3), 85-109.

Wilmot, A., Pizzey, H., Leitao, S., Hasking, P., & Boyes, M. (2022). Growing up with dyslexia: Child and parent perspectives on school struggles, self-esteem, and mental health. *Dyslexia: the Journal of the British Dyslexia Association, 09*, 09.

Yao, X., Liu, C., Xin, W., & Chen, X. (2022). Much more than just being present: Participation of children with intellectual disabilities in mainstream schools. *International Journal of Developmental Disabilities*.

Zablocki, M., & Krezmien, M. P. (2013). Drop-Out Predictors Among Students With High-Incidence Disabilities: A National Longitudinal and Transitional Study 2 Analysis. *Journal of Disability Policy Studies, 24*(1), 53-64.

Zafiriadis, K., Livaditis, M., Xenitidis, K., Diamanti, M., Tsatalmpasidou, E., Sigalas, I., & Polemikos, N. (2005). Social and psychological characteristics of Greek secondary school students with learning difficulties. *J Adolesc, 28*(6), 741-752.

Zendarski, N., Guo, S., Sciberras, E., Efron, D., Quach, J., Winter, L., Bisset, M., Middeldorp, C. M., & Coghill, D. (2020). Examining the Educational Gap for Children with ADHD and Subthreshold ADHD. *Journal of Attention Disorders*, 1087054720972790.

Zigmond, N., & Thornton, H. (1985). Follow-up of postsecondary age learning disabled graduates and drop-outs. *Learning Disabilities Research, 1*(1), 50-55.

Zoccolillo, M., & Rogers, K. (1991). Characteristics and outcome of hospitalized adolescent girls with conduct disorder. *J Am Acad Child Adolesc Psychiatry, 30*(6), 973-981.

## No data on absenteeism in autistic children and adolescents

Ahrens, K., DuBois, D. L., Lozano, P., & Richardson, L. P. (2010). Naturally acquired mentoring relationships and young adult outcomes among adolescents with learning disabilities. *Learning Disabilities Research & Practice, 25*(4), 207-216.

Allen, J. L., Morris, A., & Chhoa, C. Y. (2016). Callous-unemotional (CU) traits in adolescent boys and response to teacher reward and discipline strategies. *Emotional and Behavioural Difficulties, 21*(3), 329-342.

Almsbhieen, M. M. (2016). Early Intervention Services, Importance and Availability Extent from Workers Point of View, Who Serves in the Intellectual Disability Centers in Ma'an, Jordan. *International Education Studies, 9*(10), 216-230.

Amai, K. (2021). Adolescents’ mental health problems, teacher support, and school adaptation: A qualitative analysis based on the trajectory equifinality model. *International Journal of School & Educational Psychology*.

Arora, S., Goodall, S., Viney, R., & Einfeld, S. (2020). Societal cost of childhood intellectual disability in Australia. *Journal of Intellectual Disability Research, 64*(7), 524-537.

August, G. J., Realmuto, G. M., MacDonald, A. W., 3rd, Nugent, S. M., & Crosby, R. (1996). Prevalence of ADHD and comorbid disorders among elementary school children screened for disruptive behavior. *J Abnorm Child Psychol, 24*(5), 571-595.

Awaad, Y., Michon, A. M., Minarik, S., & Rizk, T. (2009). Levetiracetam in Tourette syndrome: A randomized double blind, placebo controlled study. *Journal of Pediatric Neurology, 7*(3), 257-263.

Bain, S. K., & Pelletier, K. A. (1999). Social and behavioral differences among a predominantly African American preschool sample. *Psychology in the Schools, 36*(3), 249-259.

Bartnick, W. M., & Parkay, F. W. (1991). A Comparative-Analysis of the Holding Power of General and Exceptional Education-Programs. *Remedial and Special Education, 12*(5), 17-22.

Baumbusch, J., & Lloyd, J. E. V. (2022). Research Priority Setting with Parents of Students with Learning Exceptionalities and Disabilities. *British Journal of Special Education, 49*(2), 209-229.

Beaton, M. C., Codina, G. N., & Wharton, J. C. (2021). Decommissioning normal: COVID-19 as a disruptor of school norms for young people with learning disabilities. *British Journal of Learning Disabilities, 2*, 02.

Berg, I., Butler, A., Franklin, J., Hayes, H., Lucas, C., & Sims, R. (1993). DSM-III-R disorders, social factors and management of school attendance problems in the normal population. *J Child Psychol Psychiatry, 34*(7), 1187-1203.

Bethune, K. S., & Kiser, A. (2017). Doing More With Less: Innovative Program Building in ABA and Special Education in a Rural Setting. *Rural Special Education Quarterly, 36*(1), 25-30.

Bishara, S. (2016). Self-regulated math instructions for pupils with learning disabilities. *Cogent Education, 3*(1).

Bishara, S., & Kaplan, S. (2016). Executive Functioning and Figurative Language Comprehension in Learning Disabilities. *World Journal of Education, 6*(2), 20-32.

Bitsika, V., & Sharpley, C. F. (2014). Understanding, Experiences, and Reactions to Bullying Experiences in Boys with an Autism Spectrum Disorder. *Journal of Developmental and Physical Disabilities, 26*(6), 747-761.

Blackman, J. A., & Gurka, M. J. (2007). Developmental and behavioral comorbidities of asthma in children. *J Dev Behav Pediatr, 28*(2), 92-99.

Blakeley-Smith, A., Reaven, J., Ridge, K., & Hepburn, S. (2012). Parent-child agreement of anxiety symptoms in youth with autism spectrum disorders. *Research in Autism Spectrum Disorders, 6*(2), 707-716.

Blanchard, L. T., Gurka, M. J., & Blackman, J. A. (2006). Emotional, developmental, and behavioral health of American children and their families: a report from the 2003 National Survey of Children's Health. *Pediatrics, 117*(6), e1202-1212.

Blasi, V., Baglio, G., Baglio, F., Canevini, M. P., & Zanette, M. (2017). Movement cognition and narration of the emotions treatment versus standard speech therapy in the treatment of children with borderline intellectual functioning: a randomized controlled trial. *BMC Psychiatry, 17*(1), 146.

Bond, E. C., & Oliphant, R. Y. K. (2018). Pervasive Refusal Syndrome in Autistic Spectrum Disorder. *Case Rep Psychiatry, 2018*, 5049818.

Bortes, C., Nilsson, K., & Strandh, M. (2022). Associations between children's diagnosed mental disorders and educational achievements in Sweden. *Scandinavian Journal of Public Health*, 14034948221089056.

Bruefach, T., & Reynolds, J. R. (2022). Social isolation and achievement of students with learning disabilities. *Social Science Research, 104*, 102667.

Bussing, R., Mason, D. M., Bell, L., Porter, P., & Garvan, C. (2010). Adolescent outcomes of childhood attention-deficit/hyperactivity disorder in a diverse community sample. *J Am Acad Child Adolesc Psychiatry, 49*(6), 595-605.

Carbonneau, R., Boivin, M., Brendgen, M., Nagin, D., & Tremblay, R. E. (2016). Comorbid Development of Disruptive Behaviors from age 1(1/2) to 5 Years in a Population Birth-Cohort and Association with School Adjustment in First Grade. *J Abnorm Child Psychol, 44*(4), 677-690.

Chen, W., Epstein, A., Toner, M., Murphy, N., Rudaizky, D., & Downs, J. (2022). Enabling successful life engagement in young people with ADHD: new components beyond adult models of recovery. *Disability & Rehabilitation*, 1-13.

Coxe, S., Sibley, M. H., & Becker, S. P. (2020). Presenting problem profiles for adolescents with ADHD: differences by sex, age, race, and family adversity. *Child & Adolescent Mental Health, 17*, 17.

Crawley, S. H., Lynch, P., & Vannest, K. (2006). The use of self-monitoring to reduce off-task behavior and cross-correlation examination of weekends and absences as an antecedent to off-task behavior. *Child & Family Behavior Therapy, 28*(2), 29-48.

Dababnah, S., Kim, I., Wang, Y., & Reyes, C. (2021). Brief report: Impact of the covid-19 pandemic on asian american families with children with developmental disabilities. *Journal of Developmental and Physical Disabilities*.

Dabrowski, J., King, J., Edwards, K., Yates, R., Heyman, I., Zimmerman-Brenner, S., & Murphy, T. (2017). Group interventions for children with tourette syndrome: a 12 month follow up study of a randomised controlled trial comparing comprehensive behavioural intervention and psycho-education. *Archives of disease in childhood, 102*.

Dawson-McClure, S., Calzada, E., Huang, K. Y., Kamboukos, D., Rhule, D., Kolawole, B., Petkova, E., & Brotman, L. M. (2015). A population-level approach to promoting healthy child development and school success in low-income, urban neighborhoods: impact on parenting and child conduct problems. *Prev Sci, 16*(2), 279-290.

de Vries, M., Verdam, M. G., Prins, P. J., Schmand, B. A., & Geurts, H. M. (2018). Exploring possible predictors and moderators of an executive function training for children with an autism spectrum disorder. *Autism, 22*(4), 440-449.

Dever, B. V., Kamphaus, R. W., Dowdy, E., Raines, T. C., & Distefano, C. (2013). Surveillance of middle and high school mental health risk by student self-report screener. *West J Emerg Med, 14*(4), 384-390.

Dickinson, H., Smith, C., Yates, S., & Tani, M. The importance of social supports in education: survey findings from students with disability and their families during COVID-19. *Disability & Society*.

Duke, R. E., Torty, C., Okorie, U., Kim, M. J., Eneli, N., Edadi, U., Burton, K., Tann, C., & Bowman, R. (2021). Pattern of comorbidities in school-aged children with cerebral palsy in Cross River State, Nigeria. *Bmc Pediatrics, 21*(1).

Dwarakanath, S., Hegde, A., Ketan, J., Chandrajit, P., Yadav, R., Keshav, K., Sampath, S., Pal, P. K., & Reddy, Y. C. (2017). "I swear, I can't stop it!" - A case of severe Tourette's syndrome treated with deep brain stimulation of anteromedial globus pallidus interna. *Neurol India, 65*(1), 99-102.

Fleming, M., McLay, J. S., Clark, D., King, A., Mackay, D. F., Minnis, H., & Pell, J. P. (2021). Educational and health outcomes of schoolchildren in local authority care in Scotland: A retrospective record linkage study. *PLoS Medicine / Public Library of Science, 18*(11), e1003832.

Flores, J., Caqueo-Urizar, A., Lopez, V., & Acevedo, D. (2022). Symptomatology of attention deficit, hyperactivity and defiant behavior as predictors of academic achievement. *BMC Psychiatry, 22*(1), 61.

Flynn, S., Hastings, R. P., McNamara, R., Gillespie, D., Randell, E., Richards, L., & Taylor, Z. (2019). Who's Challenging Who?: a co-produced approach for training staff in learning disability services about challenging behaviour. *Tizard learning disability review, 24*(4), 192‐199.

Franke, K. B. (2016). The Dual Factor Model and the Emerging Disability Paradigm: Looking beyond Deficits of Youth with Autism Spectrum Disorder. *Communique, 44*(7), 16-18.

Furusho, J., Matsuzaki, K., Ichihashi, I., Satoh, H., Yamaguchi, K., & Kumagai, K. (2001). Alleviation of sleep disturbance and repetitive behavior by a selective serotonin re-uptake inhibitor in a boy with Asperger's syndrome. *Brain Dev, 23*(2), 135-137.

Garcia Murillo, L., Ramos-Olazagasti, M. A., Klein, R. G., Mannuzza, S., & Castellanos, F. X. (2019). Correlates of nicotine dependence in men with childhood attention-deficit/hyperactivity disorder: a 33-year follow-up. *Atten Defic Hyperact Disord, 11*(2), 183-189.

Gates, A. I. (1936). Failure in reading and social maladjustment. *Journal of the National Education Association, 25*, 205-206.

Genova, H. M., Arora, A., & Botticello, A. L. (2021). Effects of School Closures Resulting From COVID-19 in Autistic and Neurotypical Children. *Frontiers in Education, 6*.

Glomb, N. K., Buckley, L. D., Minskoff, E. D., & Rogers, S. (2006). The Learning Leaders Mentoring Program for Children with ADHD and Learning Disabilities. *Preventing School Failure, 50*(4), 31-35.

Greig, A., & MacKay, T. (2005). Asperger's Syndrome and cognitive behaviour therapy: New applications for educational psychologists. *Educational and Child Psychology, 22*(4), 4-15.

Groenewald, C. B., Tham, S. W., & Palermo, T. M. (2020). Impaired School Functioning in Children With Chronic Pain: A National Perspective. *Clinical Journal of Pain, 36*(9), 693-699.

Güleç-Aslan, Y. (2013). A Training Programme for a Teacher Working with a Student with ASD: An Action Research. *Educational Sciences: Theory and Practice, 13*(4), 2229-2246.

Hagborg, W. J. (1998). School membership among students with learning disabilities and nondisabled students in a semirural high school. *Psychology in the Schools, 35*(2), 183-188.

Hamilton, L. G., Kelly, L., & Mesa, S. (2023). "I'm able to function better when I know there's a beginning and an end time": Autistic adolescents' experiences of lockdowns during the COVID-19 pandemic. *Autism & Developmental Language Impairments, 8*.

Harardottir, S., Juliusdottir, S., & Gumundsson, H. S. (2015). Understanding resilience in learning difficulties: Unheard voices of secondary school students. *Child & Adolescent Social Work Journal, 32*(4), 351-358.

Hido, M., & Shehu, I. (2010). Beyond Stigmatization of Children with Difficulties in Learning. *US-China Education Review, 7*(10), 70-77.

Hill, C., Keville, S., & Ludlow, A. K. Inclusivity for children with autism spectrum disorders: Parent's reflections of the school learning environment versus home learning during COVID-19. *International Journal of Developmental Disabilities*.

Hinshaw, S. P., Owens, E. B., Wells, K. C., Kraemer, H. C., Abikoff, H. B., Arnold, L. E., Conners, C. K., Elliott, G., Greenhill, L. L., Hechtman, L., Hoza, B., Jensen, P. S., March, J. S., Newcorn, J. H., Pelham, W. E., Swanson, J. M., Vitiello, B., & Wigal, T. (2000). Family processes and treatment outcome in the MTA: negative/ineffective parenting practices in relation to multimodal treatment. *J Abnorm Child Psychol, 28*(6), 555-568.

Hinze-Pifer, R., & Sartain, L. (2018). Rethinking Universal Suspension for Severe Student Behavior. *Peabody Journal of Education, 93*(2), 228-243.

Hirsch, S. E., McDaniel, S. C., La Salle, T., & Walker, A. C. (2021). Instructional Management for Students with Emotional and Behavioral Disorders in Remote Learning Environments. *Intervention in School and Clinic, 57*(2), 78-86.

Hoffmann, M. S., McDaid, D., Salum, G. A., Silva-Ribeiro, W., Ziebold, C., King, D., Gadelha, A., Miguel, E. C., Mari, J. D., Rohde, L. A., Pan, P. M., Bressan, R. A., Mojtabai, R., & Evans-Lacko, S. (2021). The impact of child psychiatric conditions on future educational outcomes among a community cohort in Brazil. *Epidemiology and Psychiatric Sciences, 30*.

Hysing, M., Askeland, K. G., La Greca, A. M., Solberg, M. E., Breivik, K., & Sivertsen, B. (2019). Bullying Involvement in Adolescence: Implications for Sleep, Mental Health, and Academic Outcomes. *J Interpers Violence*, 886260519853409.

Kamal, M., & Bener, A. (2009). Factors contributing to school failure among school children in very fast developing Arabian Society. *Oman Med J, 24*(3), 212-217.

Kandemir, H., Kilic, B. G., Ekinci, S., & Yuce, M. (2014). An evaluation of the quality of life of children with ADHD and their families. *Anadolu Psikiyatri Dergisi-Anatolian Journal of Psychiatry, 15*(3), 265-271.

Kawabe, K., Horiuchi, F., & Ueno, S.-i. (2019). Combination treatment with guanfacine extended release and blonanserin for Tourette's syndrome comorbid with attention deficit hyperactivity disorder. *Psychiatry and Clinical Neurosciences, 73*(12), 762-763.

Keane, E., Aldridge, F. J., Costley, D., & Clark, T. (2012). Students with autism in regular classes: a long-term follow-up study of a satellite class transition model. *International Journal of Inclusive Education, 16*(10), 1001-1017.

Keilitz, I., & Dunivant, N. (1986). The relationship between learning disability and juvenile delinquency: Current state of knowledge. *RASE: Remedial & Special Education, 7*(3), 18-26.

Kern, L., Evans, S. W., Lewis, T. J., State, T. M., Mehta, P. D., Weist, M. D., Wills, H. P., & Gage, N. A. (2021). Evaluation of a Comprehensive Assessment-Based Intervention for Secondary Students With Social, Emotional, and Behavioral Problems. *Journal of Emotional and Behavioral Disorders, 29*(1), 44-55.

Kester, K. R., & Lucyshyn, J. M. (2019). Co-creating a school-based Facing Your Fears anxiety treatment for children with autism spectrum disorder: A model for school psychology. *Psychology in the Schools, 56*(5), 824-839.

Khasakhala, E., & Galava, P. (2016). Relationship between Teachers' Perception of Causes of Challenging Behaviour and the Choice of Management Strategies among Learners with Autistic Spectrum Disorders. *Journal of Education and Practice, 7*(2), 80-87.

Lahey, B. B., Goodman, S. H., Waldman, I. D., Bird, H., Canino, G., Jensen, P., Regier, D., Leaf, P. J., Gordon, R., & Applegate, B. (1999). Relation of age of onset to the type and severity of child and adolescent conduct problems. *J Abnorm Child Psychol, 27*(4), 247-260.

Langberg, J. M., Epstein, J. N., Urbanowicz, C. M., Simon, J. O., & Graham, A. J. (2008). Efficacy of an organization skills intervention to improve the academic functioning of students with attention-deficit/hyperactivity disorder. *School Psychology Quarterly, 23*(3), 407-417.

Langenbach, T., Sponlein, A., Overfeld, E., Wiltfang, G., Quecke, N., Scherbaum, N., Melchers, P., & Hebebrand, J. (2010). Axis I comorbidity in adolescent inpatients referred for treatment of substance use disorders. *Child Adolesc Psychiatry Ment Health, 4*, 25.

Langone, J., & et al. (1992). Co-Operative School Programmes in the State of Georgia: An Analysis. *International Journal of Rehabilitation Research, 15*(1), 69-74.

Larco, A., Carrillo, J., Chicaiza, N., Yanez, C., & Lujan-Mora, S. (2021). Moving beyond Limitations: Designing the Helpdys App for Children with Dyslexia in Rural Areas. *Sustainability, 13*(13).

Lee, M.-S., Lee, H.-Y., & Kim, S.-H. (2008). Relapse of tic symptoms in a patient diagnosed with obssesive-compulsive disorder and treated with high-dose paroxetine. *Journal of Child and Adolescent Psychopharmacology, 18*(3), 305-306.

Linton, A. C., Germundsson, P., Heimann, M., & Danermark, B. (2013). Teachers' social representation of students with Asperger diagnosis. *European Journal of Special Needs Education, 28*(4), 392-412.

Lodygowska, E., & Czepita, D. A. (2012). School phobia in children with dyslexia. *Ann Acad Med Stetin, 58*(1), 66-70.

Lollar, D. J., Hartzell, M. S., & Evans, M. A. (2012). Functional difficulties and health conditions among children with special health needs. *Pediatrics, 129*(3), e714-722.

Looff, D. H. (1990). Recognizing and treating attention deficit disorders in chemically dependent adolescents. *Journal of Adolescent Chemical Dependency, 1*(1), 5-30.

Lopez, M. F. (1996). Children with Attention Deficit Hyperactivity Disorder and Emotional or Behavioral Disorders in Primary Grades: Inappropriate Placement in the Learning Disability Category. *Education and Treatment of Children, 19*(3), 286-299.

Lynch, R. J., Kistner, J. A., & Allan, N. P. (2014). Distinguishing among disruptive behaviors to help predict high school graduation: does gender matter? *J Sch Psychol, 52*(4), 407-418.

MacDonald, V. M., & Achenbach, T. M. (1999). Attention problems versus conduct problems as 6-year predictors of signs of disturbance in a national sample. *J Am Acad Child Adolesc Psychiatry, 38*(10), 1254-1261.

Magiati, I., Ong, C., Lim, X. Y., Tan, J. W., Ong, A. Y., Patrycia, F., Fung, D. S., Sung, M., Poon, K. K., & Howlin, P. (2016). Anxiety symptoms in young people with autism spectrum disorder attending special schools: Associations with gender, adaptive functioning and autism symptomatology. *Autism, 20*(3), 306-320.

Mammarella, I. C., Ghisi, M., Bomba, M., Bottesi, G., Caviola, S., Broggi, F., & Nacinovich, R. (2016). Anxiety and Depression in Children With Nonverbal Learning Disabilities, Reading Disabilities, or Typical Development. *J Learn Disabil, 49*(2), 130-139.

Mann, R. L. (2006). Effective Teaching Strategies for Gifted/Learning-Disabled Students With Spatial Strengths. *Special Issue: New voices in gifted education., 17*(2), 112-121.

Manti, E., Scholte, E. M., & Van Berckelaer-Onnes, I. A. (2011). Development of children with autism spectrum disorders in special needs education schools in the Netherlands: a three-year follow-up study. *European Journal of Special Needs Education, 26*(4), 411-427.

Matthews, L. K., Wall, K. H., Hoffman, Y., Pantale, A., & De Martinis, J. (2019). The unheard voices of transition: The experiences of four female young adults with ASD as they prepare to graduate. *The High School Journal, 102*(4), 283-296.

Mattison, R. E., & Schneider, J. (2009). First-Year Effectiveness on School Functioning of a Self-Contained ED Middle School. *Behavioral Disorders, 34*(2), 60-71.

Merga, M. K. (2020). "Fallen through the cracks": Teachers' perceptions of barriers faced by struggling literacy learners in secondary school. *English in Education, 54*(4), 371-395.

Mikami, A. Y., Cox, D. J., Davis, M. T., Wilson, H. K., Merkel, R. L., & Burket, R. (2009). Sex differences in effectiveness of extended-release stimulant medication among adolescents with attention-deficit/hyperactivity disorder. *J Clin Psychol Med Settings, 16*(3), 233-242.

Mills, J. R., & Clarke, M. (2017). Dyslexia and the Need for Teacher Training: A Collaborative Three-Pronged Approach between a University and a Community Partner. *Leadership and Research in Education, 4*(1), 77-89.

Minami, K., & Horikawa, E. (2020). Social anxiety tendency and autism spectrum disorder in Japanese Adolescence. *Pediatrics International, 12*, 12.

Mineur, T. (2015). Swedish education in upper secondary schools for students with intellectual disabilities: from a student perspective. *Research and Practice in Intellectual and Developmental Disabilities, 2*(2), 165-179.

Miniarikova, E., Audras-Torrent, L., Berard, M., Peries, M., Picot, M. C., Munir, K., & Baghdadli, A. (2023). Adaptive behaviors and related factors in children and adolescents with autism spectrum disorder: Report from ELENA cohort. *Journal of Psychiatric Research, 163*, 43-54.

Minkos, M. L., Chafouleas, S. M., Bray, M. A., & LaSalle, T. P. (2018). Brief Report: A Preliminary Investigation of a Mindful Breathing Intervention to Increase Academic Engagement in an Alternative Educational Setting. *Behavioral Disorders, 43*(4), 436-443.

Miyake, Y., Lum Min, S. A., Yamataka, A., & Keijzer, R. (2023a). Educational and mental health outcomes in school-aged children with anorectal malformations: A case-control cohort study. *Journal of Pediatric Surgery, 58*(5), 844-848.

Miyake, Y., Lum Min, S. A., Yamataka, A., & Keijzer, R. (2023b). The impact of intestinal atresia on educational and mental health outcomes in school-aged children: A case-control cohort study. *Pediatric Surgery International, 39*(1), 86.

Mohamad, N. B., Lee, K. Y., Mansor, W., Mahmoodin, Z., Fadzal, C. W., & Amirin, S. (2015). EEG-based time and spatial interpretation of activation areas for relaxation and words writing between poor and capable dyslexic children. *Conf Proc IEEE Eng Med Biol Soc, 2015*, 4757-4760.

Mohammed, A. M. H. (2016). The Cognitive Abilities and Skills of Children Who Suffer from Attention Deficit and Hyperactivity Disorder (ADHD) in Kuwait State. *Journal of Education and Practice, 7*(17), 149-155.

Naheed, A., Islam, M. S., Hossain, S. W., Ahmed, H. U., Uddin, M. M. J., Tofail, F., Hamadani, J. D., Hussain, A., & Munir, K. (2019). Burden of major depressive disorder and quality of life among mothers of children with autism spectrum disorder in urban bangladesh. *Autism Res, 24*, 24.

Neal, S., & Frederickson, N. (2016). ASD transition to mainstream secondary: a positive experience? *Educational Psychology in Practice, 32*(4), 355-373.

Orsmond, G. I., Munsell, E. G. S., & Coster, W. J. (2020). The Status of Service and Support Provision for Diploma-Track High School Students on the Autism Spectrum. *Journal of Special Education Leadership, 33*(2), 90-105.

Oshie, T. (2020). Community play therapy for encounter with diverse children. *Person-Centered and Experiential Psychotherapies, 19*(3), 243-250.

Paavonen, E. J., Almqvist, F., Tamminen, T., Moilanen, I., Piha, J., Rasanen, E., & Aronen, E. T. (2002). Poor sleep and psychiatric symptoms at school: an epidemiological study. *Eur Child Adolesc Psychiatry, 11*(1), 10-17.

Pallisera, M., Fullana, J., Puyalto, C., & Vila, M. (2016). Changes and challenges in the transition to adulthood: views and experiences of young people with learning disabilities and their families. *European Journal of Special Needs Education, 31*(3), 391-406.

Park, S., Como, P. G., Cui, L., & Kurlan, R. (1993). The early course of the Tourette's syndrome clinical spectrum. *Neurology, 43*(9), 1712-1715.

Pikkarainen, M. T., Hakala, J. T., & Kykyri, V.-L. (2021). Why Did They Leave School? A Self Determination Theory Perspective into Narratives of Finnish Early School Leavers. *International Journal of Educational Psychology, 10*(1), 48-72.

Polimanti, R., & Gelernter, J. (2017). Widespread signatures of positive selection in common risk alleles associated to autism spectrum disorder. *PLoS Genet, 13*(2), e1006618.

Pronk, S., Mulder, E., van den Berg, G., Stams, G., Popma, A., & Kuiper, C. (2020). Differences between adolescents who do and do not successfully complete their program within a non-residential alternative education facility. *Children and Youth Services Review Vol 109 2020, ArtID 104735, 109*.

Pumpuang, W., Phuphaibul, R., Orathai, P., & Putdivarnichapong, W. (2012). Effectiveness of a Collaborative Home-School Behavior Management Program for Parents and Teachers of Children with Attention Deficit Hyperactivity Disorder. *Pacific rim international journal of nursing research, 16*(2), 138‐153.

Radzeviciene, L., Miliuniene, L., Baturaite, A., Hoogerwerf, E. J., Charvatis, A., Bader, M., Grudeva, P., & Oliv, T. (2019). Analysis of Cognition Structure of Students Having Emotional, Behavioral and Learning Difficulties: Aspect of International Experience. *Social Welfare Interdisciplinary Approach, 9*(1), 158-167.

Rajhans, P., Sagar, R., Patra, B. N., Bhargava, R., & Kabra, S. K. (2021). Psychiatric Morbidity and Behavioral Problems in Children and Adolescents with Bronchial Asthma. *Indian Journal of Pediatrics, 24*, 24.

Ramasamy, R. (1996). Post-high school employment: A follow-up of Apache Native American youth. *Journal of Learning Disabilities, 29*(2), 174-179.

Ramey, D. M. (2020). Punitive versus Medicalized Responses to Childhood Behavior Problems and High School Graduation. *Sociological Perspectives, 63*(2), 201-227.

Rattaz, C., Munir, K., Michelon, C., Picot, M. C., Baghdadli, A., & group, E. s. (2020). School Inclusion in Children and Adolescents with Autism Spectrum Disorders in France: Report from the ELENA French Cohort Study. *J Autism Dev Disord, 50*(2), 455-466.

Riley, R. W. (1996). Improving the reading and writing skills of America's students. *Learning Disability Quarterly, 19*(2), 67-69.

Roberts, D. J. (1975). A survey of 235 Salford handicapped school leavers for the years 1970, 1971 and 1972, compared with 235 non-handicappped school leavers for the same years. *Public Health, 89*(5), 207-211.

Ross, P., & Randolph, J. (2016). Differences between Students with and without ADHD on Task Vigilance under Conditions of Distraction. *Journal of Educational Research and Practice, 4*(1), 1-10.

Rushton, S., Giallo, R., & Efron, D. (2019). ADHD and emotional engagement with school in the primary years: Investigating the role of student-teacher relationships. *Br J Educ Psychol, 26*, 26.

Rushton, S., Giallo, R., & Efron, D. (2020). ADHD and emotional engagement with school in the primary years: Investigating the role of student-teacher relationships. *British Journal of Educational Psychology, 90 Suppl 1*, 193-209.

Saele, R. G., Sorlie, T., Nergard-Nilssen, T., Ottosen, K. O., Goll, C. B., & Friborg, O. (2016). Demographic and psychological predictors of grade point average (GPA) in North-Norway: a particular analysis of cognitive/school-related and literacy problems. *Educational Psychology, 36*(10), 1886-1907.

Sahoo, T., Gulla, K. M., Kumar, N., & Malhi, P. (2019). Case of Extreme Trichotillomania in a Child with Comorbidities. *Journal of Clinical and Diagnostic Research, 13*(7), Sd1-Sd2.

Sanderson, K. A., & Goldman, S. E. Understanding the Characteristics and Predictors of Student Involvement in IEP Meetings. *Journal of Special Education*.

Scahill, L., Bearss, K., Lecavalier, L., Smith, T., Swiezy, N., Aman, M. G., Sukhodolsky, D. G., McCracken, C., Minshawi, N., Turner, K., Levato, L., Saulnier, C., Dziura, J., & Johnson, C. (2016). Effect of Parent Training on Adaptive Behavior in Children With Autism Spectrum Disorder and Disruptive Behavior: Results of a Randomized Trial. *J Am Acad Child Adolesc Psychiatry, 55*(7), 602-609 e603.

Scanlon, G., & Doyle, A. (2021). Transition Stories: Voices of School Leavers with Intellectual Disabilities. *British Journal of Learning Disabilities, 49*(4), 456-466.

Schnitzer, G., Andries, C., & Lebeer, J. (2007). Usefulness of cognitive intervention programmes for socio-emotional and behaviour problems in children with learning disabilities. *Journal of Research in Special Educational Needs, 7*(3), 161-171.

Shen, L., Wang, C. X., Tian, Y., Chen, J. J., Wang, Y., & Yu, G. J. (2021). Effects of Parent-Teacher Training on Academic Performance and Parental Anxiety in School-Aged Children With Attention-Deficit/Hyperactivity Disorder: A Cluster Randomized Controlled Trial in Shanghai, China. *Frontiers in Psychology, 12*.

Sibley, M. H., Coxe, S. J., Campez, M., Morley, C., Olson, S., Hidalgo-Gato, N., Gnagy, E., Greiner, A., Coles, E. K., Page, T., & Pelham, W. E. (2018). High versus Low Intensity Summer Treatment for ADHD Delivered at Secondary School Transitions. *J Clin Child Adolesc Psychol, 47*(2), 248-265.

Sibley, M. H., Ortiz, M., Gaias, L. M., Reyes, R., Joshi, M., Alexander, D., & Graziano, P. (2021). Top problems of adolescents and young adults with ADHD during the COVID-19 pandemic. *Journal of Psychiatric Research, 136*, 190-197.

Sprick, J. T., Bouck, E. C., Berg, T. R., & Coughlin, C. (2020). Attendance and Specific Learning Disability Identification: A Survey of Practicing School Psychologists. *Learning Disabilities Research & Practice, 35*(3), 139-149.

Stark, I., Liao, P. W., Magnusson, C., Lundberg, M., Rai, D., Lager, A., & Nordstrom, S. I. Qualification for upper secondary education in individuals with autism without intellectual disability: Total population study, Stockholm, Sweden. *Autism*.

Sun, L., Semovski, V., & Stewart, S. L. A Study of Risk Factors Predicting School Disruption in Children and Youth Living in Ontario. *Canadian Journal of School Psychology*.

Taanila, A., Yliherva, A., Kaakinen, M., Moilanen, I., & Ebeling, H. (2011). An epidemiological study on Finnish school-aged children with learning difficulties and behavioural problems. *Int J Circumpolar Health, 70*(1), 59-71.

Takagi, M. J., Lubman, D. I., & Yucel, M. (2008). Interpreting neuropsychological impairment among adolescent inhalant users: two case reports. *Acta Neuropsychiatr, 20*(1), 41-43.

Taylor, J. L., & DaWalt, L. S. (2017). Brief Report: Postsecondary Work and Educational Disruptions for Youth on the Autism Spectrum. *J Autism Dev Disord, 47*(12), 4025-4031.

Thompson, T., Davis, S., Janusz, J., Frith, E., Pyle, L., Howell, S., Boada, R., Wilson, R., & Tartaglia, N. (2022). Supporting students with sex chromosome aneuploidies in educational settings: Results of a nationwide survey. *Journal of School Psychology, 93*, 28-40.

Tremblay, P. (2013). Comparative outcomes of two instructional models for students with learning disabilities: Inclusion with co-teaching and solo-taught special education. *Journal of Research in Special Educational Needs, 13*(4), 251-258.

Ulu, H., & Akyol, H. (2016). The Effects of Repetitive Reading and PQRS Strategy in the Development of Reading Skill. *Eurasian Journal of Educational Research*(63), 225-242.

Usami, M., Iwadare, Y., Watanabe, K., Ushijima, H., Kodaira, M., Okada, T., Sasayama, D., Sugiyama, N., & Saito, K. (2015). A case-control study of the difficulties in daily functioning experienced by children with depressive disorder. *J Affect Disord, 179*, 167-174.

Van Praag, L., Van Caudenberg, R., & Orozco, M. (2018). Age is more than just a number! The role of age and maturity in the processes leading to early school leaving in Flanders (Belgium). *British Educational Research Journal, 44*(4), 557-572.

Verma, S., & Agrawal, R. (2021). Psychotropic Medication Adherence in Children and Adolescents. *Southern Medical Journal, 114*(7), 388-394.

Verrier, D., Halton, S., & Robinson, M. (2020). Autistic traits, adolescence, and anti-social peer pressure. *Current Issues in Personality Psychology, 8*(2), 131-138.

Wallin, J. E. W. (1922). A study of the industrial record of children assigned to public school classes for mental defectives, and legislation in the interest of defectives. *The Journal of Abnormal Psychology and Social Psychology, 17*(2), 120-131.

Wei, X., Wagner, M., Hudson, L., Yu, J. W., & Javitz, H. (2016). The Effect of Transition Planning Participation and Goal-Setting on College Enrollment Among Youth With Autism Spectrum Disorders. *Remedial and Special Education, 37*(1), 3-14.

Whelan, M., McGillivray, J., & Rinehart, N. J. (2023). Using Life Course Theory to Explore the Association Between Autistic Traits, Child, Family, and School Factors and the Successful Transition to Secondary School. *Journal of Autism & Developmental Disorders, 06*, 06.

Willoughby, M. T., Murray, D., Kuhn, L. J., Cavanaugh, A. M., & LaForett, D. R. (2022). Incorporating callous-unemotional behaviors into school-based research. *School Psychologist, 7*, 07.

Yngve, M., Lidström, H., Ekbladh, E., & Hemmingsson, H. (2018). Which students need accommodations the most, and to what extent are their needs met by regular upper secondary school? A cross-sectional study among students with special educational needs. *European Journal of Special Needs Education, 34*(3), 327-341.

Zenenga, A., Phillips, J., Nyashanu, M., & Ekpenyong, M. S. (2023). Exploring the Impact of Animal Involvement in the Learning Experiences of Learners Mainly with Autism in the English West Midlands Region: A Qualitative Study. *Journal of Education, 203*(1), 10-17.

## Type of publication

1986 More on Tourette syndrome and school phobia. *Am J Psychiatry, 143*(2), 265-266.

Armbruster, B., & Howe, C. E. (1985). Educators Team up to Help Students Learn. *NASSP Bulletin, 69*(479), 82-86.

Bain, A. (1988). Issues in the Suspension and Exclusion of Disruptive Students. *Australasian Journal of Special Education, 12*(2), 19-24.

Bakwin, H. (1965). Learning problems and school phobia. *Pediatr Clin North Am, 12*(4), 995-1014.

Barbaresi, W. J., Katusic, S. K., Colligan, R. C., Weaver, A. L., Mrazek, D. A., & Jacobsen, S. J. (2003). School dropout and grade retention among children with ADHD: A population-based birth cohort study. *Pediatric Research, 53*(4), 65a-66a.

Bellini, B., Arruda, M., Cescut, A., Saulle, C., Persico, A., Carotenuto, M., Gatta, M., Nacinovich, R., Piazza, F. P., Termine, C., Tozzi, E., Lucchese, F., & Guidetti, V. (2013). Headache and comorbidity in children and adolescents. *J Headache Pain, 14*, 79.

Berg, I. (1992). Absence from school and mental health. *Br J Psychiatry, 161*, 154-166.

Black, L. I., & Zablotsky, B. (2018). Chronic School Absenteeism Among Children With Selected Developmental Disabilities: National Health Interview Survey, 2014-2016. *Natl Health Stat Report*(118), 1-7.

Carney, J., & Salorio, C. (2016). Translating Assessment into Effective School Services for Students with Epilepsy. *Journal of Pediatric Epilepsy, 06*(01), 031-036.

Casoli-Reardon, M., Rappaport, N., Kulick, D., & Reinfeld, S. (2012). Ending School Avoidance. *Educational Leadership, 70*(2), 50-55.

Cassel, R. N. (1988). Fitness, school dropouts, and delinquency: The health crisis in our schools. *College Student Journal, 22*(2), 192-198.

Chaban, P. (2010). Partnering Research and Practice in High Schools. *Education Canada, 50*(5), 0.

Closson, M., & Rogers, K. M. (2007). Educational needs of youth in the juvenile justice system. *Mental Health Needs of Young Offenders: Forging Paths toward Reintegration and Rehabilitation*, 229-240.

Cobb, B., Sample, P. L., Alwell, M., & Johns, N. R. (2006). Cognitive-behavioral interventions, dropout, and youth with disabilities - A systematic review. *Remedial and Special Education, 27*(5), 259-275.

Colvin, M. K., Reesman, J., & Glen, T. (2022). Reforming learning disorder diagnosis following COVID-19 educational disruption. *Nature reviews Psychology, 1*(5), 251-252.

Colvin, M. K. M., Reesman, J., & Glen, T. (2022). The impact of COVID-19 related educational disruption on children and adolescents: An interim data summary and commentary on ten considerations for neuropsychological practice. *Clinical Neuropsychologist, 36*(1), 45-71.

Cromer, B. A., McLean, C. S., & Heald, F. P. (1992). A critical review of comprehensive health screening in adolescents: Psychosocial screening. *Journal of Adolescent Health, 13*(2, Suppl), 52-57.

Ctri. (2018). *School-based Programme for Emotional and Behavioural problems in adolescents*. https://www.cochranelibrary.com/central/doi/10.1002/central/CN-01897565/full

Daniels, H. (2018). Welcome to issue 23(2) of the journal. *Emotional and Behavioural Difficulties, 23*(2), 109-110.

deBettencourt, L. U., & Zigmond, N. (1990). The Learning Disabled Secondary School Dropout: What Teachers Should Know. What Teachers Can Do. *Teacher Education and Special Education, 13*(1), 17-20.

Denton, C. A., & Al Otaiba, S. (2011). Teaching Word Identification to Students with Reading Difficulties and Disabilities. *Focus Except Child, 2011*(7), 254245149.

Deshler, D. D. (2005). Adolescents with learning disabilities: Unique challenges and reasons for hope. *Learning Disability Quarterly, 28*(2), 122-124.

Deshpande, S., Ostermeyer, B., Lokhande, A., Abid, H., & Shah, A. A. (2017). Managing Adult Attention-Deficit/Hyperactivity Disorder: To Treat or Not To Treat? *Psychiatric Annals, 47*(6), 315-321.

Donnelly, J. P. (2021). In response to "Decommissioning normal: COVID-19 as a disruptor of school norms for young people with learning disabilities". *British Journal of Learning Disabilities, 49*(4), 403-405.

DuPaul, G. J. (2018). Promoting Success Across School Years for Children With Attention-Deficit/Hyperactivity Disorder: Collaborative School-Home Intervention. *J Am Acad Child Adolesc Psychiatry, 57*(4), 231-232.

Dvorsky, M. R., & Langberg, J. M. (2016). A Review of Factors that Promote Resilience in Youth with ADHD and ADHD Symptoms. *Clin Child Fam Psychol Rev, 19*(4), 368-391.

Elliott, J. G., & Place, M. (2019). Practitioner Review: School refusal: developments in conceptualisation and treatment since 2000. *J Child Psychol Psychiatry, 60*(1), 4-15.

Ensminger, E. E., & Dangel, H. L. (1992). The Foxfire Pedagogy - a Confluence of Best Practices for Special-Education. *Focus on Exceptional Children, 24*(7), 1-16.

Evans, I. M., & Matthews, A. K. (1992). A behavioral approach to the prevention of school dropout: conceptual and empirical strategies for children and youth. *Prog Behav Modif, 28*, 219-249.

Fibert, P., Relton, C., Peasgood, T., & Daley, D. (2018). Protocol for the STAR (Sheffield Treatments for ADHD) project: an internal pilot study assessing the feasibility of the Trials within Cohorts (TwiCs) design to test the effectiveness of interventions for children with ADHD. *Pilot Feasibility Stud, 4*, 61.

Finn, C. A., Heath, N. L., Petrakos, H., & McLean-Heywood, D. (2002). Work-in-progress: A comparison of school service models for children at risk for emotional and behavioral disorders. *Canadian Journal of School Psychology, 17*(2), 61-68.

Freeman, J., & Simonsen, B. (2015). Examining the Impact of Policy and Practice Interventions on High School Dropout and School Completion Rates: A Systematic Review of the Literature. *Review of Educational Research, 85*(2), 205-248.

Freeman, J., Yell, M. L., Shriner, J. G., & Katsiyannis, A. (2019). Federal Policy on Improving Outcomes for Students With Emotional and Behavioral Disorders: Past, Present, and Future. *Behavioral Disorders, 44*(2), 97-106.

Fulton, M., Sciberras, E., Efron, D., Oberklaid, F., & Hiscock, H. (2009). Behavioural sleep intervention in school aged children with attention deficit hyperactivity disorder (ADHD). *Sleep and biological rhythms, 7*, A33‐.

Futenma, K., Takaesu, Y., Komada, Y., Shimura, A., Okajima, I., Matsui, K., Tanioka, K., & Inoue, Y. (2023). Delayed sleep-wake phase disorder and its related sleep behaviors in the young generation. *Frontiers in psychiatry Frontiers Research Foundation, 14*, 1174719.

Galili-Weisstub, E., & Segman, R. H. (2003). Attention deficit and hyperactivity disorder: review of genetic association studies. *Isr J Psychiatry Relat Sci, 40*(1), 57-66.

Gau, S. S. (2011). Childhood trajectories of inattention symptoms predicting educational attainment in young adults. *Am J Psychiatry, 168*(11), 1131-1133.

Glascoe, F. P. (1999). Using parents' concerns to detect and address developmental and behavioral problems. *J Soc Pediatr Nurs, 4*(1), 24-35.

Goddard, H. H. (1914). Social problems. *Feeble-mindedness: Its causes and consequences.*, 1-20.

Gonzalez, L., & Cramer, E. (2013). Class Placement and Academic and Behavioral Variables as Predictors of Graduation for Students with Disabilities. *Journal of Urban Learning, Teaching, and Research, 9*, 112-123.

Grandin, T. (1990). Needs of high functioning teenagers and adults with autism: Tips from a recovered autistic. *Focus on Autistic Behavior, 5*(1), 16.

Gray, K., Borland, R., Hu, N., Melvin, G., Hastings, R., Totsika, V., Heyne, D., & Tonge, B. J. (2019). School Non-Attendance in Australian Children with Autism Spectrum Disorder. *Journal of Intellectual Disability Research, 63*(7), 859-860.

Guyer, B. P. (1974). The Montessori approach for the elementary-age LD child. *Academic Therapy, 10*(2), 187-192.

Harris, K. I., & Sholtis, S. D. (2016). Companion Angels on a Leash: Welcoming Service Dogs Into Classroom Communities for Children With Autism. *Childhood Education, 92*(4), 263-275.

Hastings, R., Gray, K., Heyne, D., Totsika, V., Tonge, B. J., Ashford, L., & Melvin, G. (2019). School Attendance in Australian School Students with an Intellectual Disability. *Journal of Intellectual Disability Research, 63*(7), 859-859.

Heyman, E. (2004). Learning deficits may account for cases of school phobia. *Am Fam Physician, 70*(6), 1032.

Heyne, D. (2022). Developmental Issues Associated with Adolescent School Refusal and Cognitive-Behavioral Therapy Manuals A Practitioner Review. *Zeitschrift Fur Kinder-Und Jugendpsychiatrie Und Psychotherapie, 50*(6), 471-494.

Hogue, A., Bobek, M., & Evans, S. W. (2016). Changing Academic Support in the Home for Adolescents With Attention-Deficit/Hyperactivity Disorder: A Family-Based Clinical Protocol for Improving School Performance. *Cognitive and Behavioral Practice, 23*(1), 14-30.

Hopkins, A. F., & Hughes, M.-a. (2015). Individualized Health Care Plans. *Young Exceptional Children, 19*(2), 33-44.

Hu, X. Y., Wilczynski, S. M., Ma, Y. Q., & Jin, N. International use of N=1 research design with secondary students with disabilities. *Psychology in the Schools*.

Hunter, L. (2003). School psychology: a public health framework III. Managing disruptive behavior in schools: the value of a public health and evidence-based perspective. *Journal of School Psychology, 41*(1), 39-59.

Kahn, J. H. (1966). About school phobia. *Midwife Health Visit, 2*(11), 464-469.

Kawabe, K. (2019). School refusal and Internet addiction in adolescents with Autism Spectrum Disorder. *Journal of Behavioral Addictions, 8*, 45-45.

Kearney, C. A. (2003). Bridging the gap among professionals who address youths with school absenteeism: Overview and suggestions for consensus. *Professional Psychology-Research and Practice, 34*(1), 57-65.

Kearney, C. A., & Benoit, L. (2022). Child and Adolescent Psychiatry and Underrepresented Youth With School Attendance Problems: Integration With Systems of Care, Advocacy, and Future Directions. *Journal of the American Academy of Child & Adolescent Psychiatry, 61*(10), 1208-1210.

Keegan, N. F., Brigham, F. J., Cardellichio, J. M., & Brigham, M. M. (2005). Advocating for students with learning disabilities and attention deficit hyperactivity disorder in public schools. *J Long Term Eff Med Implants, 15*(4), 389-400.

Kiani, C., Otero, K., Taufique, S., & Ivanov, I. (2018). Chronic Absenteeism: A Brief Review of Causes, Course and Treatment. *Adolescent Psychiatry, 8*(3), 214-230.

Knage, F. S. Beyond the school refusal/truancy binary: engaging with the complexities of extended school non-attendance. *International Studies in Sociology of Education*.

Kortering, L. J. (2009). School Completion Issues in Special Education. *Exceptionality, 17*(1), 1-4.

Kortering, L. J., & Christenson, S. (2009). Engaging Students in School and Learning: The Real Deal for School Completion. *Exceptionality, 17*(1), 5-15.

Kumar, P., & Agrawal, N. (2019). Learning Disabled and Their Education in India. *Human Arenas, 2*(2), 228-244.

Kumazaki, H., Muramatsu, T., Yoshikawa, Y., Matsumoto, Y., Ishiguro, H., Sumiyoshi, T., Mimura, M., & Kikuchi, M. (2019). Comedic experience with two robots aided a child with autism spectrum disorder to realize the importance of nonverbal communication. *Psychiatry Clin Neurosci, 73*(7), 423.

Leduc, K., Tougas, A. M., Robert, V., & Boulanger, C. (2022). School Refusal in Youth: A Systematic Review of Ecological Factors. *Child Psychiatry & Human Development*.

Linet, L. S. (1986). More on Tourette Syndrome and School Phobia - Reply. *American Journal of Psychiatry, 143*(2), 266-266.

Loe, I. M., & Feldman, H. M. (2007). Academic and educational outcomes of children with ADHD. *J Pediatr Psychol, 32*(6), 643-654.

Luey, J. (2014). Participating, Navigating and Succeeding with Autism Spectrum Disorder in the Ontario Postsecondary Education System. *College Quarterly, 17*(4), 15.

Luna, J., Gladson, A., & Looney, G. (2018). A study of the effectiveness of literacy interventions on middle and high school students. *Dissertation Abstracts International Section A: Humanities and Social Sciences, 79*(3-A(E)), No-Specified.

McGee, A. (2011). Skills, standards, and disabilities: How youth with learning disabilities fare in high school and beyond. *Economics of Education Review, 30*(1), 109-129.

Mcgee, J. J., & Lin, F. Y. (2017). Providing a supportive alternative education environment for at-risk students. *Preventing School Failure, 61*(2), 181-187.

Mcloughlin, J. A., Nall, M., & Petrosko, J. (1985). Allergies and Learning-Disabilities. *Learning Disability Quarterly, 8*(4), 255-260.

Melkevik, O., Nilsen, W., Evensen, M., Reneflot, A., & Mykletun, A. (2016). Internalizing Disorders as Risk Factors for Early School Leaving: A Systematic Review. *Adolescent Research Review, 1*(3), 245-255.

Melvin, G. A., & Gordon, M. S. (2019). Antidepressant Medication: Is It a Viable and Valuable Adjunct to Cognitive-Behavioral Therapy for School Refusal? *Cognitive and Behavioral Practice, 26*(1), 107-118.

Melvin, G. A., Heyne, D., Gray, K. M., Hastings, R. P., Totsika, V., Tonge, B. J., & Freeman, M. M. (2019). The Kids and Teens at School (KiTeS) Framework: An Inclusive Bioecological Systems Approach to Understanding School Absenteeism and School Attendance Problems. *Frontiers in Education, 4*.

Minahan, J., & Rappaport, N. (2012). Anxiety in students A hidden culprit in behavior issues. *Phi Delta Kappan, 94*(4), 34-39.

Mirza, H., Roberts, E., Al-Belushi, M., Al-Salti, H., Al-Hosni, A., Jeyaseelan, L., & Al-Adawi, S. (2018). School Dropout and Associated Factors Among Omani Children with Attention-Deficit Hyperactivity Disorder: A Cross-Sectional Study. *J Dev Behav Pediatr, 39*(2), 109-115.

Morrison, G. M., & Cosden, M. A. (1997). Risk, resilience, and adjustment of individuals with learning disabilities. *Learning Disability Quarterly, 20*(1), 43-60.

Morrison, K. (2007). Implementation of Assistive Computer Technology: A Model for School Systems. *International Journal of Special Education, 22*(1), 83-95.

Muljana, P. S., & Luo, T. (2019). Factors Contributing to Student Retention in Online Learning and Recommended Strategies for Improvement: A Systematic Literature Review. *Journal of Information Technology Education-Research, 18*, 19-57.

Nachane, B. S. N. (2016). Clinical Profile of Children Presenting with School Refusal : A Retrospective Analysis. *Indian Journal of Psychiatry, 58*(5), S100-S100.

Nct. (2021a). Investigation of Strategies to Reduce the Impact of the Relative Age Effect in Kindergarten. *https://clinicaltrials.gov/show/NCT05142826*.

Nct. (2021b). RUBIES in Educational Settings. *https://clinicaltrials.gov/show/NCT05093686*.

Norbeck, C. L. (2017). What's hope got to do with it? A narrative inquiry into the hope levels of high school students diagnosed with soft disabilities. *Dissertation Abstracts International Section A: Humanities and Social Sciences, 78*(4-A(E)), No-Specified.

Ntr. (2018). Increasing adolescents cognitive functioning through physical education. *http://www.who.int/trialsearch/Trial2.aspx?TrialID=NTR7098*.

Nuske, H. J., McGhee Hassrick, E., Bronstein, B., Hauptman, L., Aponte, C., Levato, L., Stahmer, A., Mandell, D. S., Mundy, P., Kasari, C., & Smith, T. (2019). Broken bridges-new school transitions for students with autism spectrum disorder: A systematic review on difficulties and strategies for success. *Autism, 23*(2), 306-325.

O'Brien, J. (1982). School problems: school phobia and learning disabilities. *Psychiatr Clin North Am, 5*(2), 297-307.

O'Regan, F. (2013). Persistent disruptive behaviours becoming persistent school exclusion and the role of attention-deficit/hyperactivity disorder. *European Child & Adolescent Psychiatry, 22*, S130-S130.

Office of the Surgeon, G. (2021). *US Department of Health and Human Services. Publications and Reports of the Surgeon General*.

Osher, D., Morrison, G., & Bailey, W. (2003). Exploring the relationship between student mobility and dropout among students with emotional and behavioral disorders. *Special Issue: Student mobility: How some children get left behind., 72*(1), 79-91.

Perera, D. M., & Wheeler, M. (2021). Mental Health Informed Educators: Facilitating Student Academic Success. *New Educator, 17*(3), 281-304.

Perry, J. L. (1986). Field Initiated Research: A Field Based Study of the Effects of an Educational Program on School Adjustment of Mildly Handicapped Secondary School Students. Final Report. *Wake County Public School System, Raleigh, N.C.*

Pollard, N. L. (1998). Development of social interaction skills in preschool children with autism: A review of the literature. *Child & Family Behavior Therapy, 20*(2), 1-16.

Rauch-Elnekave, H. (1994). Teenage motherhood: its relationship to undetected learning problems. *Adolescence, 29*(113), 91-103.

Riddle, M. A., Hardin, M. T., Ort, S. I., Leckman, J. F., & Cohen, D. J. (1988). Behavioral symptoms in Tourette's syndrome. *Tourette's syndrome and tic disorders: Clinical understanding and treatment.*, 151-162.

Rosenheck, R. A., & Scivoletto, S. (2016). Childhood Mental Illness and Adult Homelessness: Social Policy and Long-Term Community-Based Psychiatric Care. *J Am Acad Child Adolesc Psychiatry, 55*(11), 923-924.

Sansosti, F. J., Cimera, R. E., Koch, L. C., & Rumrill, P. (2017). Strategies for ensuring positive transition for individuals with attention-deficit/hyperactivity disorder. *Journal of Vocational Rehabilitation, 47*(2), 149-157.

Schlozman, S. C., & Schlozman, V. R. (2000). Chaos in the Classroom: Looking as ADHD. *Educational Leadership, 58*(3), 28-33.

Schwenn, J. O. (1991). Learning disabilities. *Understanding students with high incidence exceptionalities: Categorical and noncategorical perspectives.*, 30-67.

Sciberras, E., Efron, D., Patel, P., Mulraney, M., Lee, K. J., Mihalopoulos, C., Engel, L., Rapee, R. M., Anderson, V., Nicholson, J. M., Schembri, R., & Hiscock, H. (2019). Does the treatment of anxiety in children with Attention-Deficit/Hyperactivity Disorder (ADHD) using cognitive behavioral therapy improve child and family outcomes? Protocol for a randomized controlled trial. *BMC Psychiatry, 19*(1), 359.

Shaw, S. R. (2010). Rescuing Students from the Slow Learner Trap. *Principal Leadership, 10*(6), 12-16.

Shores, R. E., Gunter, P. L., Denny, R. K., & Jack, S. L. (1993). Classroom Influences on Aggressive and Disruptive Behaviors of Students with Emotional and Behavioral-Disorders. *Focus on Exceptional Children, 26*(2), 1-10.

Skuse, D. (2010). Social cognition and school exclusion. *Mental capital and wellbeing.*, 829-838.

State, T. M., Simonsen, B., Hirn, R. G., & Wills, H. (2019). Bridging the Research-to-Practice Gap Through Effective Professional Development for Teachers Working With Students With Emotional and Behavioral Disorders. *Behavioral Disorders, 44*(2), 107-116.

Sullivan, A. L., & Sadeh, S. (2016). Does the Empirical Literature Inform Prevention of Dropout among Students with Emotional Disturbance? A Systematic Review and Call to Action. *Exceptionality, 24*(4), 251-262.

Test, D. W., Fowler, C. H., White, J., Richter, S., & Walker, A. (2009). Evidence-Based Secondary Transition Practices for Enhancing School Completion. *Exceptionality, 17*(1), 16-29.

Thastum, M., Johnsen, D. B., Silverman, W. K., Jeppesen, P., Heyne, D. A., & Lomholt, J. J. (2019). The Back2School modular cognitive behavioral intervention for youths with problematic school absenteeism: study protocol for a randomized controlled trial. *Trials, 20*(1), 29.

Toldson, I. A. (2011). Editor's comment: How Black boys with disabilities end up in honors classes while others without disabilities end up in special education. *Journal of Negro Education, 80*(4), 439-443.

Tong, Y., Wang, S., Cao, L., Zhu, D., Wang, F., Xie, F., Zhang, X., Wang, G., & Su, P. (2023). School dropouts related to mental disorders: A systematic review and meta-analysis. *Asian Journal of Psychiatry, 85*, 103622.

Tonge, B. J., & Silverman, W. K. (2019). Reflections on the Field of School Attendance Problems: For the Times They Are a-Changing? *Cognitive and Behavioral Practice, 26*(1), 119-126.

Totsika, V., Hastings, R., Dutton, Y., Worsley, A., Melvin, G., Gray, K., Tonge, B. J., & Heyne, D. (2019, Jul). *Rate and Type of School Non-Attendance in Children and Young People with Autism Spectrum Disorder in the Uk* [Meeting Abstract]. Journal of Intellectual Disability Research,

Tyson, M., & Valman, H. B. (1981). ABC of 1 to 7. School failure. *Br Med J (Clin Res Ed), 283*(6299), 1102-1103.

Vance, H. B. (1977). Trends in Secondary Curriculum-Development. *Academic Therapy, 13*(1), 29-35.

Watling, R. (2004). Helping them out: The role of teachers and healthcare professionals in the exclusion of pupils with special educational needs. *Emotional & Behavioural Difficulties, 9*(1), 8-27.

Wender, E. H. (1995). Attention-deficit hyperactivity disorders in adolescence. *J Dev Behav Pediatr, 16*(3), 192-195.

Weyandt, L. L., & Swentosky, A. (2013). Attention deficit hyperactivity disorder. *The neuropsychology of psychopathology.*, 59-74.

Wilkins, J., & Bost, L. W. (2015). Re-Engaging School Dropouts with Emotional and Behavioral Disorders. *Phi Delta Kappan, 96*(4), 52-56.

Williams, J. M. (2016). From the perspective of a person with autism spectrum disorder. *Autism spectrum disorder.*, 475-483.

Winters, C. A. (1997). Learning disabilities, crime, delinquency, and special education placement. *Adolescence, 32*(126), 451-462.

Zendarski, N., Sciberras, E., Mensah, F., & Hiscock, H. (2016). A longitudinal study of risk and protective factors associated with successful transition to secondary school in youth with ADHD: prospective cohort study protocol. *BMC Pediatr, 16*, 20, Article 20.

Zulauf-McCurdy, C. A., Coxe, S. J., Lyon, A. R., Aaronson, B., Ortiz, M., & Sibley, M. H. (2021). Study protocol of a randomised trial of Summer STRIPES: a peer-delivered high school preparatory intervention for students with ADHD. *BMJ Open, 11*(8), e045443.

## Not retrieved

Manset-Williamson, G., & Washburn, S. (2002). Administrators' Perspectives of the Impact of Mandatory Graduation Qualifying Examinations for Students with Learning Disabilities. *Journal of Special Education Leadership, 15*(2), 49-59.

Tutto, D. N. (1956). Maladjustment among adolescents. *Vidya Bhawan Studies, 5*, 105-111.
